# A Combination of *Psidium guajava* and *Seriphium plumosum* Modulates Glucose Metabolism and Reduces Hepatic GLUT2 Expression Through Additive-to-Synergistic Interactions

**DOI:** 10.3390/nu18172920

**Published:** 2026-09-06

**Authors:** Nokukhanya Thembane, Siboniso Sithole, Sphamandla Hlatshwayo, Phikelelani Siphosethu Ngubane, Mlungisi Ngcobo, Nceba Gqaleni

**Affiliations:** 1 Department of Biomedical Sciences, Faculty of Applied and Health Sciences, Mangosuthu University of Technology, Durban 4026, South Africa; 2 Sub-Discipline of Traditional Medicine, School of Medicine, College of Health Sciences, University of KwaZulu-Natal, Durban 4041, South Africa; siboniso121@gmail.com (S.S.); sphanox@yahoo.com (S.H.); ngcobom3@ukzn.ac.za (M.N.); 3 Department of Human Physiology, School of Medicine, College of Health Sciences, University of KwaZulu-Natal, Durban 4000, South Africa; ngubanep1@ukzn.ac.za; 4 Africa Health Research Institute, Nelson R. Mandela School of Medicine, Durban 4001, South Africa

**Keywords:** *Psidium guajava*, *Seriphium plumosum*, type 2 diabetes mellitus, metabolic syndrome, glucose uptake, GLUT2, bioactive compounds, functional foods, glycaemic control, synergistic interactions

## Abstract

**Background:** Type 2 diabetes mellitus (T2DM) remains a major global health challenge, prompting interest in plant-based interventions with multi-target metabolic effects. This study evaluated the individual and combined effects of *Psidium guajava* (PG) and *Seriphium plumosum* (SP) on hepatic and skeletal muscle glucose metabolism. **Methods:** HepG2 hepatocytes and differentiated C2C12 myotubes were treated with PG, SP, and a 1:1 PG+SP combination. Cell viability, glucose uptake, GLUT2 expression, glycogen content, and AMP levels were assessed. Interactions were evaluated using Bliss Independence, Combination Index (CI), Loewe Additivity, and Zero Interaction Potency (ZIP) models. **Results:** SP exhibited minimal cytotoxicity, whereas PG induced concentration-dependent reductions in HepG2 viability. The PG+SP combination maintained higher viability than PG alone and demonstrated additive-to-synergistic interactions (CI = 0.51–0.89). In HepG2 cells, all treatments significantly enhanced glucose uptake relative to controls (*p* < 0.05), with the combination achieving approximately 94% of the metformin response. The combination also significantly reduced GLUT2 expression, with the greatest effect observed at 125 + 125 µg/mL (0.77 ± 0.14-fold, *p* < 0.0001). In C2C12 myotubes, PG, SP, and PG+SP significantly increased glucose uptake and glycogen accumulation (*p* < 0.05). SP increased AMP levels at lower concentrations, whereas the PG+SP combination reduced AMP levels below control values. Synergy analyses consistently indicated concentration- and endpoint-dependent interactions ranging from additivity to synergy. **Conclusions:** The PG+SP combination co-ordinately modulated glucose uptake, glycogen storage, GLUT2 expression, and AMP-related energy status across hepatic and skeletal muscle models. These findings provide mechanistic support for further investigation of this botanical combination as a potential multi-target strategy for T2DM and metabolic dysfunction.

## 1. Introduction

Type 2 diabetes mellitus (T2DM) affects over 537 million adults worldwide, with sub-Saharan Africa experiencing the fastest prevalence increases [1,2]. The disease arises from three interconnected defects: impaired insulin secretion, increased hepatic glucose production, and reduced peripheral glucose uptake [3,4]. The liver and skeletal muscle play complementary, non-redundant roles in glucose homeostasis: the liver contributes approximately 30% of postprandial glucose disposal and governs fasting glucose via gluconeogenesis and glycogenolysis [5,6,7], while insulin resistance disrupts this suppression, driving fasting hyperglycaemia [8]. Skeletal muscle accounts for 70–80% of insulin-stimulated glucose uptake, and impaired GLUT4 translocation reduces glucose entry into myocytes in T2DM [9,10,11]. Therapeutic strategies that target both compartments are therefore mechanistically well-founded [12]. Metformin, the first-line oral agent, activates AMPK to suppress hepatic gluconeogenesis and modestly enhance peripheral uptake [13,14,15], but causes gastrointestinal side effects in 20–30% of patients [16]; insulin therapy is effective but carries hypoglycaemia risk and cold-chain requirements that limit access in low-resource settings [15,17]. Neither agent fully restores glucose homeostasis nor addresses all three pathophysiological defects simultaneously [18], sustaining interest in plant-derived agents offering multi-target effects at lower cost [19].

### 1.1. Synergy Assessment in Polyherbal Research

Combination therapy can achieve efficacy at lower individual doses by targeting multiple pathways simultaneously [20,21]. Synergy—where the combined effect exceeds the sum of individual effects—is particularly relevant to plant extracts, which contain multiple bioactive compounds acting through complementary mechanisms [22,23]. We define additivity as a combined effect equal to the sum of individual effects (CI = 1; Bliss, Loewe, and ZIP scores near zero) and synergy as an effect exceeding that sum (CI < 1; positive Bliss, Loewe, and ZIP scores) [24,25,26,27]. This distinction has both therapeutic and toxicological relevance: synergy can enable dose reduction and multi-target efficacy in drug development [21], but the same interactions may produce unpredictable effects when herbal combinations are co-administered with conventional antidiabetic agents [19,22]. Because plant constituents can also modulate CYP450 enzymes or P-glycoprotein-mediated efflux, synergy assessment must consider pharmacokinetic as well as pharmacodynamic dimensions [22,23]. No single synergy model is definitive; each makes different assumptions about dose–response relationships, so using multiple models strengthens confidence in interaction conclusions [27,28]. The present study therefore applies four independent frameworks, namely Bliss Independence, Combination Index, Loewe Additivity, and Zero Interaction Potency, to characterise PG+SP interactions robustly.

Recent studies illustrate both the value and the limits of single-model, single-extract approaches. A 2025 study on a mulberry leaf–*Siraitia grosvenorii* combination reported synergistic enzyme inhibition (Combination Index) but relied entirely on cell-free enzymatic, metabolomic, and docking assays, without live-cell validation [29]. A study on *Holothuria tubulosa* extract measured GLUT2 protein by Western blotting and flow cytometry and linked increased GLUT2 to HNF1α upregulation in HepG2 cells, while a parallel single-extract study on *Carica papaya* assessed both HepG2 and C2C12 cells but found GLUT2 expression unchanged [30,31]. Both lines of work examined single extracts only, leaving synergistic polyherbal effects on hepatic GLUT2 regulation unexplored.

### 1.2. Candidate Plants and Transporter Biology

*Psidium guajava* (PG; Myrtaceae) has documented antidiabetic activity: aqueous extracts inhibit α-amylase, α-glucosidase, and DPP-4 in cell-free assays, with guavin C identified as a candidate DPP-4 inhibitor [32,33], consistent with its flavonoid- and phenolic-rich profile [34]. PG is among the African medicinal plants highlighted for their commercial and therapeutic importance [35]. Plant-derived polyphenol-rich extracts have been reported to exhibit concentration-dependent cytotoxicity in HepG2 cells [36]. Although PG is a polyphenol-rich plant, its effects on glucose uptake, GLUT2 expression, and metabolic signalling remain incompletely characterised, and it has not been evaluated in combination with other African medicinal plants in live-cell models.

*Seriphium plumosum* (SP; Asteraceae) is a South African plant traditionally used for metabolic disturbances [37]. SP exhibits hypoglycaemic activity and has been proposed to influence glucose-utilisation pathways, namely GLUT4-mediated glucose uptake in skeletal muscle via Akt phosphorylation [38]. Nonetheless its effects on hepatic GLUT2 expression have not been studied, a significant gap given the liver’s role in postprandial disposal and fasting hyperglycaemia.

GLUT2 and GLUT4 operate through fundamentally different mechanisms: GLUT4 is insulin-sensitive and found mainly in muscle and adipose tissue, translocating to the plasma membrane in response to insulin—a process impaired in T2DM. GLUT2, in contrast, is a high-capacity, low-affinity transporter expressed predominantly in hepatocytes and pancreatic β-cells, constitutively mediating bidirectional glucose flux and regulated primarily by glucose availability and HNF1α [39,40,41,42]. Measuring GLUT2 protein, as done here, therefore captures a distinct axis of glucose handling from skeletal-muscle GLUT4 activity [43].

### 1.3. Knowledge Gaps and Study Aims

A recent systematic review protocol highlighted the scarcity of mechanistic evidence for polyherbal T2DM combinations in African populations and proposed the theoretical potential of combining PG and SP based on their individual phytochemical profiles without direct evaluation [5]. In related work, synthetic alkyl gallates and gallotannins have been evaluated for their genotoxic, DNA-protective, and antioxidant properties in vitro [44], reinforcing the importance of mechanistic assays for phytochemical agents. To our knowledge, no study has evaluated the combined effects of PG and SP in live-cell models, quantified their interaction using multi-model synergy analysis, or assessed both hepatic and skeletal-muscle compartments in parallel. Using HepG2 cells (a well-established hepatic glucose-metabolism model expressing GLUT2 [45,46]) and C2C12 myotubes (a validated model of insulin-stimulated peripheral glucose disposal expressing GLUT4 [47,48]), we assessed: (1) the cytotoxicity of PG, SP, and their 1:1 combination in HepG2 cells, with synergy analysis across four models; (2) glucose uptake in HepG2 cells; (3) GLUT2 protein expression by ELISA; and (4) glucose uptake, glycogen content, and AMP levels in C2C12 myoblasts as indicators of cellular energy status, with synergy quantified across all endpoints.

We hypothesised that the PG+SP combination would show synergy across multiple models with reduced cytotoxicity relative to PG alone; that it would increase glucose uptake above either extract individually in both cell lines; that it would modulate GLUT2 protein expression in HepG2 cells, a mechanism distinct from the GLUT4-mediated effects previously reported for SP in muscle [38]; and that it would improve glycogen content and AMP-linked energy status in C2C12 myoblasts, endpoints not previously reported for either plant. Relative to prior work, this study provides the first live-cell (rather than cell-free or in silico) validation of a PG+SP combination, the first parallel hepatic and muscle assessment for either plant, the first GLUT2 data for SP in a hepatic context, and a four-model synergy framework exceeding the single-model approaches common in the field [27,28].

## 2. Materials and Methods

### 2.1. Plant Material and Extract Preparation

Leaves of *Psidium guajava* L. and *Seriphium plumosum* L. were collected from KwaZulu-Natal, South Africa, on 31 March 2024. Voucher specimens were identified by a qualified botanist and deposited at the Bews Herbarium, University of KwaZulu-Natal (Pietermaritzburg Campus), under accession numbers UDW23005 (*P. guajava*) and UDW23006 (*S. plumosum*). Leaves were washed under running distilled water, rinsed with deionised water, and dried in a single layer at ambient temperature (22–25 °C) in a well-ventilated, shaded room for 14 days until constant weight was achieved. Dried leaves were pulverised using a laboratory-scale hammer mill and passed through a 0.5 mm mesh sieve. The resulting powder was stored in amber-coloured, airtight glass containers at 4 °C until extraction.

Aqueous extracts were prepared by boiling 21.34 g of powdered leaf material in 2.0 L of distilled water for 45 min under reflux condensation. The decoction was cooled to room temperature, steeped overnight (12 h) with continuous orbital shaking at 120 rpm, and centrifuged at 1500× *g* for 10 min at 4 °C. The supernatant was frozen at −80 °C for 24 h and lyophilised at −50 °C under a vacuum of 0.050 mbar for 72 h using a freeze dryer. Lyophilised extracts were stored in a desiccator at −20 °C until use. Extraction yield was calculated gravimetrically as: Yield (%) = (mass of lyophilised extract/mass of starting plant material) × 100. The yields were 18.4% for *P. guajava* and 22.7% for *S. plumosum*.

### 2.2. UPLC-PDA-HRMS Phytochemical Profiling

#### 2.2.1. Sample Preparation for UPLC-PDA-HRMS Analysis

For comprehensive chemical characterisation, lyophilised extracts of *P. guajava* (PG) and *S. plumosum* (SP) were reconstituted in a 1:1 (*v*/*v*) methanol/ultra-pure water (MeOH/H_2_O) mixture at concentrations of 2.0 mg/mL and 1.5 mg/mL, respectively. Samples were vortexed for 1 min, sonicated for 5 min at room temperature, and centrifuged at 14,000× *g* for 10 min at 4 °C. The supernatant was filtered through 0.22 µm nylon syringe filters (Millex, Merck KGaA, Darmstadt, Germany) into amber high-performance liquid chromatography (HPLC) vials. All samples were prepared in triplicate and stored at 4 °C prior to analysis, with analysis completed within 24 h of preparation to ensure stability.

#### 2.2.2. UPLC-PDA-HRMS Instrumentation and Conditions

Chemical profiling was performed using an ACQUITY UPLC^®^ I-Class system (Waters Corporation, Milford, MA, USA) coupled to a photodiode array detector (PDA) and a XEVO-G2-XS QTOF mass spectrometer (Waters Corporation). Chromatographic separation was achieved on an ACQUITY UPLC^®^ BEH C18 (Waters Corporation, supplied by Microsep (Pty) Ltd., Johannesburg, South Africa), analytical column (100 mm × 2.1 mm, i.d., 1.7 µm particle size) maintained at 50 °C. The mobile phase consisted of (A) water and (B) methanol, both containing 0.1% (*v*/*v*) formic acid. The flow rate was 0.3 mL/min, and the following linear gradient was applied: 0–1 min, 97% A; 1–14 min, gradient to 100% B; 14–17 min, 100% B; 17–17.5 min, gradient to 97% A; 17.5–20 min, 97% A (equilibration). The injection volume was 5.0 µL.

The mass spectrometer was operated in both positive and negative electrospray ionisation (ESI) modes over a mass range of *m*/*z* 50–1500 Da. The capillary voltage was 2.5 kV, source temperature 120 °C, and desolvation temperature 450 °C. Desolvation gas flow was 800 L/h, with a cone gas flow of 50 L/h. Data were acquired in MS^E^ (data-independent acquisition) mode, using a low-energy collision function (4 eV) and a high-energy ramp (15–50 eV) to obtain both precursor and fragment ion information. External calibration was performed using sodium formate, and leucine-enkephalin ([M + H]^+^ = 556.2771 Da; [M − H]^−^ = 554.2615 Da) was used as a lock-mass reference at 10 µL/min for real-time mass correction, ensuring mass accuracy < 5 ppm.

#### 2.2.3. Data Processing and Compound Identification

Raw UPLC-PDA-HRMS data were acquired and processed using MassLynx™ v4.1 software (Waters Corporation). Peak detection, chromatographic alignment, and lock-mass correction were performed using the Waters_connect UNIFI™ scientific information system. Parameters for peak detection included a minimum peak intensity of 1000 counts and a peak width of 6 s. Putative compound identification was achieved by matching accurate mass ([M + H]^+^, [M − H]^−^, and adduct ions), isotopic pattern, and MS/MS fragmentation spectra against the following natural product databases: LOTUS natural products database (https://lotus.naturalproducts.net/, accessed on 1 March 2026), PubChem (https://pubchem.ncbi.nlm.nih.gov/, accessed on 1 March 2026), and the Dictionary of Natural Products (DNP, CRC Press). Only identifications with a mass error < 5 ppm and a fragment ion match score > 60% were considered as confident assignments. Compounds that could not be unequivocally identified were annotated as “tentatively identified” based on molecular formula and characteristic fragmentation patterns. Chromatographic retention times and UV-Vis spectral data (210–600 nm) from the PDA detector were also used to support identification and aid in compound class assignment.

#### 2.2.4. Rationale for UPLC-PDA-HRMS Analysis

The integration of UPLC-PDA-HRMS in this study serves two primary purposes. First, it provides comprehensive chemical characterisation of the aqueous extracts, enabling correlation of observed bioactivity with specific phytochemical constituents. Second, it ensures batch-to-batch consistency and quality control of the plant materials used in subsequent cell-based assays. This analytical approach is particularly well-suited to complex botanical extracts, as the high-resolution mass spectrometry enables confident annotation of known compounds, while the high-resolution chromatographic separation reduces ion suppression and improves detection of minor constituents. The use of both positive and negative ionisation modes maximises coverage of the chemically diverse secondary metabolites typically present in *Psidium* and *Seriphium* species.

### 2.3. Cell Culture

HepG2 human hepatoma cells and C2C12 mouse skeletal muscle myoblasts were obtained from the American Type Culture Collection (ATCC, Manassas, VA, USA). HepG2 cells were cultured in Dulbecco’s Modified Eagle Medium (DMEM) supplemented with 10% foetal bovine serum (FBS), 100 U/mL penicillin, and 100 µg/mL streptomycin. C2C12 myoblasts were cultured in DMEM containing 10% FBS and 1% penicillin-streptomycin. Both cell lines were maintained at 37 °C in a humidified atmosphere containing 5% CO_2_. Culture medium was refreshed every 48 h. Cells were passaged at 80–90% confluence using 0.25% trypsin-EDTA. For differentiation into myotubes, C2C12 myoblasts were seeded at 2 × 10^4^ cells/cm^2^ and cultured in DMEM supplemented with 2% horse serum for 5–7 days, with medium changes every 48 h. Differentiation was confirmed by microscopic observation of multinucleated myotubes, with a minimum of three nuclei per myotube considered differentiated. This morphological criterion was applied consistently across all differentiation batches prior to experimental treatments. The differentiation protocol employed is a well-established and standardised method for inducing C2C12 myotube formation, as extensively validated in the literature [45,46].

### 2.4. Cell Viability Assay in HepG2 Cells

HepG2 cells were seeded into white-walled 96-well plates at 1 × 10^4^ cells per well in 100 µL of culture medium and incubated for 24 h to allow attachment. The medium was then replaced with fresh medium containing *P. guajava* (PG), *S. plumosum* (SP), or their 1:1 combination (PG: SP = 1:1, *w*/*w*) at final concentrations of 62.5, 125, 250, and 500 µg/mL. Metformin (160 µg/mL) was included as a positive control. Untreated cells (0 µg/mL) served as the negative control (set to 100% viability). After 24 h of exposure, plates were equilibrated to room temperature for 30 min. CellTiter-Glo^®^ reagent (100 µL; Promega, Madison, WI, USA) was added to each well, and contents were mixed on an orbital shaker for 2 min to induce cell lysis, followed by a 10-min incubation at room temperature to stabilise the luminescent signal. Luminescence was measured using a SpectraMax i3x microplate reader (Molecular Devices, San Jose, CA, USA). All experiments were performed in triplicate (*n* = 3 independent biological replicates, each with three technical replicates). Cell viability was calculated as: % Viability = (RLU_sample_/RLU_Control_) × 100, where RLU_sample_ is the luminescence of treated cells and RLU_Control_ is the luminescence of untreated cells.

### 2.5. Cell Viability Assay in C2C12 Myoblasts

C2C12 myoblasts were seeded into white-walled 96-well plates at 1 × 10^4^ cells per well in 100 µL of culture medium and incubated for 24 h. Cells were then treated with PG, SP, or their 1:1 combination at concentrations of 100, 200, 300, 400, 500, 1000, 1500, 2000, 2500, and 3000 µg/mL for 24 h. Untreated cells served as the negative control (100% viability). Cell viability was measured using the CellTiter-Glo^®^ Luminescent Cell Viability Assay as described in Section 2.3. Nonlinear regression with a four-parameter logistic (sigmoidal) curve was performed using GraphPad Prism 9.0 (San Diego, CA, USA), and Pearson’s correlation coefficient (r) was calculated to assess the linearity of the dose–response relationship.

### 2.6. Glucose Uptake Assay in HepG2 Cells

HepG2 cells were seeded into 24-well plates at 2 × 10^5^ cells per well in 500 µL of culture medium and incubated for 24 h. Cells were then treated with PG alone (62.5, 125, 250 µg/mL), SP alone (62.5, 125, 250 µg/mL), or their 1:1 combination (62.5 + 62.5, 125 + 125, 250 + 250 µg/mL) for 24 h. Metformin (160 µg/mL) served as the positive control, and untreated cells served as the negative control. Concentration selection was based on preliminary cytotoxicity data showing >80% viability at these concentrations. After treatment, cells were washed twice with phosphate-buffered saline (PBS, pH 7.4) and incubated with 1 mL of glucose-free DMEM containing 5 mM glucose for 2 h at 37 °C. Aliquots of medium (20 µL) were collected at the start (0 h) and end (2 h) of the incubation period. Glucose concentration was measured using a colorimetric glucose assay kit (Abcam, Cambridge, UK) according to the manufacturer’s protocol. Absorbance was read at 570 nm using a SpectraMax i3x microplate reader. Glucose uptake was calculated as: Glucose Uptake (%) = [(Glucose_0h_ − Glucose_2h_)/Glucose_0h_] × 100, where Glucose_0h_ is the glucose concentration at time zero and Glucose_2h_ is the glucose concentration after 2 h. All experiments were performed in triplicate from three independent experiments (*n* = 3 independent biological replicates, each with two technical replicates). Data are presented as mean ± standard error of the mean (SEM).

### 2.7. GLUT2 Protein Quantification in HepG2 Cells by ELISA

HepG2 cells were seeded into 6-well plates at 5 × 10^5^ cells per well in 2 mL of culture medium and incubated for 24 h. Cells were treated with PG alone (62.5, 125, 250 µg/mL), SP alone (62.5, 125, 250 µg/mL), or their 1:1 combination (62.5 + 62.5, 125 + 125, 250 + 250 µg/mL) for 24 h. Metformin (160 µg/mL) served as the positive control, and untreated cells served as the negative control. After treatment, the medium was removed, and cells were washed twice with ice-cold PBS. Cells were lysed in 200 µL of ice-cold RIPA buffer (50 mM Tris-HCl, pH 7.4, 150 mM NaCl, 1% NP-40, 0.5% sodium deoxycholate, 0.1% SDS) supplemented with protease inhibitor cocktail (Thermo Fisher Scientific, Waltham, MA, USA). Lysates were incubated on ice for 30 min with intermittent vortexing, then centrifuged at 14,000× *g* for 15 min at 4 °C. The supernatant (protein lysate) was collected, and total protein concentration was determined using a bicinchoninic acid (BCA) protein assay kit (Thermo Fisher Scientific, Waltham, MA, USA) according to the manufacturer’s protocol. GLUT2 protein levels were quantified using a commercial GLUT2 enzyme-linked immunosorbent assay (ELISA) kit (Abcam, Cambridge, UK). Briefly, 100 µL of each protein lysate (diluted to 50 µg/mL total protein) was added to wells pre-coated with anti-GLUT2 antibody and incubated for 2 h at room temperature. Wells were washed three times with wash buffer, followed by addition of 100 µL of biotinylated detection antibody and incubation for 1 h at room temperature. After three washes, 100 µL of horseradish peroxidase (HRP)-conjugated streptavidin was added and incubated for 30 min at room temperature. Wells were washed five times, and 100 µL of tetramethylbenzidine (TMB) substrate was added. After 15 min of incubation in the dark, the reaction was stopped with 50 µL of stop solution. Absorbance was read at 450 nm using a SpectraMax i3x microplate reader. GLUT2 concentrations were interpolated from a standard curve supplied with the kit, normalised to total protein concentration, and expressed as fold change relative to the untreated control. All experiments were performed in triplicate (*n* = 3 independent biological replicates). Data are presented as mean ± SD.

### 2.8. Glucose Uptake Assay in C2C12 Myotubes

Differentiated C2C12 myotubes were generated by culturing confluent myoblasts in DMEM supplemented with 2% horse serum for 5–7 days, with medium replacement every 48 h. Differentiation was confirmed microscopically by the formation of elongated multinucleated myotubes containing at least three nuclei per cell. Myotubes were then treated for 24 h with PG (244, 488, and 976 µg/mL), SP (243, 486, and 972 µg/mL), or their corresponding 1:1 combinations (244 + 243, 488 + 486, and 976 + 972 µg/mL). These concentrations were selected based on the cytotoxicity profile established in Section 2.4, with the highest concentrations corresponding to the upper limit of the sub-cytotoxic range (viability ≥ 80%) and the lower concentrations representing two-fold and four-fold dilutions. Insulin (100 nM) served as the positive control for glucose uptake and glycogen synthesis, and untreated cells served as the negative control. Glucose uptake was measured as described in Section 2.5, except that differentiated C2C12 myotubes cultured in 24-well plates were used instead of HepG2 cells. The incubation period for glucose uptake was 2 h. Glucose utilisation was calculated as the percentage of glucose consumed from the medium over the incubation period. All experiments were performed in triplicate (*n* = 3 independent biological replicates), and data are presented as mean ± SEM.

### 2.9. Glycogen Content Assay in C2C12 Myotubes

Differentiated C2C12 myotubes were treated as described in Section 2.7. Glycogen content was measured using a commercial fluorescence-based glycogen assay kit (Abcam, Cambridge, UK). Following treatment, cells were washed twice with PBS and lysed in 200 µL of glycogen assay buffer provided with the kit. Lysates were heated at 95 °C for 5 min to inactivate endogenous enzymes and centrifuged at 10,000× *g* for 5 min at 4 °C. The supernatants were collected, and 10 µL of each sample was transferred to a 96-well black plate, followed by the addition of 50 µL hydrolysis buffer. After incubation at 37 °C for 30 min, 50 µL development buffer was added and the plate was incubated for a further 30 min at 37 °C. Fluorescence was measured at Ex/Em = 535/590 nm using a SpectraMax i3x microplate reader (Molecular Devices, San Jose, CA, USA). Glycogen content was interpolated from a standard curve supplied with the kit and expressed as relative fluorescence units (RFU). All experiments were performed in triplicate (*n* = 3 independent biological replicates), and data are presented as mean ± SEM.

### 2.10. AMP Quantification in C2C12 Myotubes

Differentiated C2C12 myotubes were treated as described in Section 2.7. Cellular AMP levels were measured using a commercial AMP assay kit (Abcam, Cambridge, UK). Following treatment, cells were washed twice with PBS and lysed in 200 µL AMP assay buffer. Lysates were centrifuged at 10,000× *g* for 5 min at 4 °C, and the supernatants were collected. Aliquots of 50 µL were transferred to a 96-well black plate and mixed with 50 µL AMP reaction mixture. The plate was incubated at 37 °C for 30 min in the dark. Fluorescence was measured at Ex/Em = 535/587 nm using a SpectraMax i3x microplate reader. AMP concentrations were interpolated from a standard curve and expressed as relative units (RU). All experiments were performed in triplicate (*n* = 3 independent biological replicates), and data are presented as mean ± SEM.

### 2.11. Synergy Analysis

To quantify potential synergistic interactions between PG and SP, synergy was assessed using four independent, mathematically distinct methods. The use of multiple models strengthens confidence in interaction conclusions, as each model makes different assumptions about dose–response relationships [27,28].

#### 2.11.1. Combination Index (CI)

CI values were calculated using the Chou-Talalay method [23] with CompuSyn v2.0 (ComboSyn, Inc., Paramus, NJ, USA). This method is based on the median-effect principle, which assumes that the dose–response relationship follows a median-effect equation: fa/fu = (D/Dm)^m^, where fa is the fraction affected, fu is the fraction unaffected, D is the dose, Dm is the median-effect dose, and m is the Hill coefficient. The Combination Index is calculated as: CI = (D1/Dx1) + (D2/Dx2), where D1 and D2 are the doses of PG and SP in combination that produce a given effect, and Dx1 and Dx2 are the doses of each agent alone that produce the same effect. The method assumes mutually non-exclusive drug interactions. According to this model, CI < 1 indicates synergy, CI = 1 indicates additivity, and CI > 1 indicates antagonism. CI values were interpreted as follows: 0.90–1.10 = additive, 0.80–0.90 = mild synergy, 0.60–0.80 = moderate synergy, and <0.60 = strong synergy.

#### 2.11.2. Bliss Independence

Bliss synergy scores were calculated using the equation: Bliss Score = Observed effect − Expected effect, where Expected = Effect_PG + Effect_SP − Control [24]. This model assumes that the two agents act independently and that their effects are multiplicative rather than additive on the fractional scale. For HepG2 cell viability measured on a percentage scale (0–100%), scores > +10 were classified as synergy, scores between −10 and +10 as additivity, and scores < −10 as antagonism. For GLUT2 protein levels and C2C12 parameters measured on a fractional scale (0–1), scores > +0.10 were classified as synergy, scores between −0.10 and +0.10 as additivity, and scores < −0.10 as antagonism. These thresholds were selected based on established conventions in the literature [24,27,28].

#### 2.11.3. Loewe Additivity

Loewe synergy scores were estimated from fixed-ratio dose–response modelling using single-agent and combination response data. This model assumes that an agent cannot synergise with itself; the expected combination effect is calculated by solving for the dose pair that would produce the same effect if the agents were identical. Mathematically, the Loewe additivity model is expressed as: (d1/D1) + (d2/D2) = 1, where d1 and d2 are the doses of the two agents in combination, and D1 and D2 are the doses of each agent alone that produce the same effect. Positive Loewe scores indicate that the observed effect exceeds the predicted additive effect (synergy), values near zero indicate additivity, and negative scores indicate antagonism [25].

#### 2.11.4. Zero Interaction Potency (ZIP)

ZIP scores were estimated using fixed-ratio interaction modelling. This model compares the observed combination response to the expected response under the assumption of zero interaction, accounting for both dose–response curves and potency shifts. The ZIP model combines the concepts of dose–response curve shifting and potency modulation to estimate expected combination effects. Scores > 10 were classified as synergy, scores between −10 and +10 as additivity, and scores < −10 as antagonism [26]. For all synergy analyses, interaction classification was considered robust when at least three of the four models agreed on the direction of interaction (synergy, additivity, or antagonism) [27,28].

#### 2.11.5. Rationale for Multi-Model Synergy Assessment

The use of four independent synergy models (Bliss, CI, Loewe, and ZIP) reflects current best practice in drug combination research, as each model operates on distinct mathematical assumptions regarding dose–response relationships and expected combined effects [27,28]. Convergence across models strengthens confidence in interaction classifications, reducing the likelihood of model-dependent artefacts [49]. This multi-model approach is particularly relevant to phytopharmacology, where complex mixtures of bioactive compounds may produce non-linear, multi-target effects that challenge single-model interpretations [22,23]. The present study therefore employs all four frameworks to provide a robust and reproducible characterisation of PG+SP interactions across multiple metabolic endpoints.

### 2.12. Statistical Analysis

All data are presented as mean ± standard deviation (SD) or mean ± standard error of the mean (SEM), as indicated. Statistical comparisons among treatment groups were performed using one-way analysis of variance (ANOVA) followed by Dunnett’s multiple-comparison test against the untreated control. This approach was applied consistently across cell viability, glucose uptake, GLUT2 protein expression, glycogen content, and AMP datasets. Pearson’s correlation coefficient (r) was calculated to assess dose–response relationships in C2C12 viability experiments. Nonlinear regression analyses were performed using a four-parameter logistic model in GraphPad Prism 9.0 (GraphPad Software, San Diego, CA, USA). Statistical significance was accepted at *p* < 0.05. Statistical power was limited by the sample size; consequently, findings with borderline significance (*p*-values between 0.05 and 0.10) were interpreted as trends rather than definitive effects. Emphasis was placed on the consistency of findings across independent experiments rather than on individual statistical comparisons.

## 3. Results

### 3.1. Phytochemical Profiling of P. guajava and S. plumosum Aqueous Extracts

**Figure 1:**
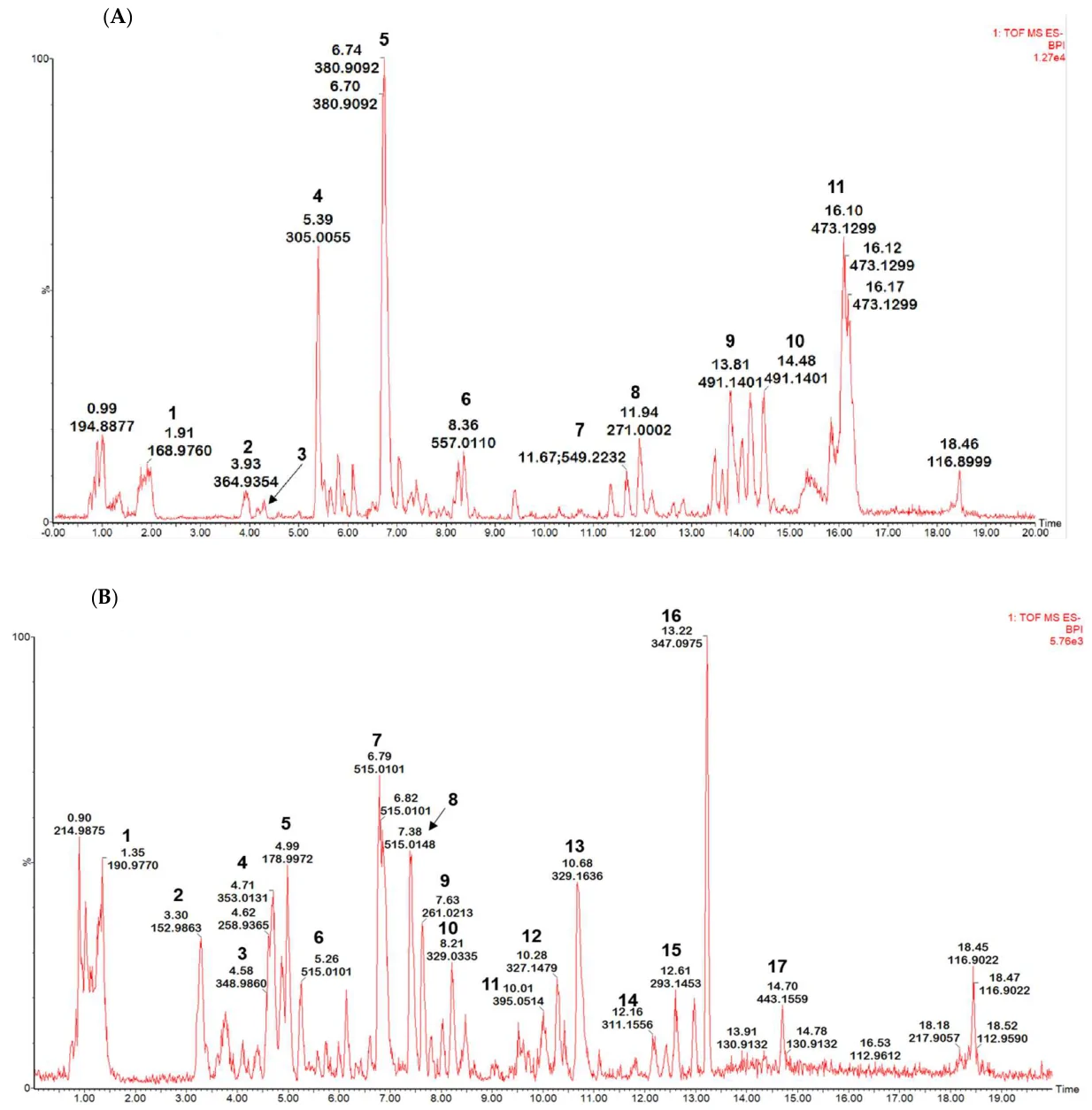
BPI UPLC-HRMS chromatograms of (**A**) *P. guajava* (VJM-I-150B) aqueous extract in negative ESI mode and (**B**) *S. plumosum* (VJM-I-150A) aqueous extract in negative ESI mode. Peaks are numbered according to Tables S1 and S2, respectively.

The chemical composition of the aqueous extracts of *P. guajava* (PG) and *S. plumosum* (SP) was characterised using UPLC-PDA-HRMS in both positive and negative electrospray ionisation (ESI) modes. The base peak intensity (BPI) chromatograms revealed complex and distinct phytochemical profiles for each extract, with negligible background interference observed in blank runs (Figure 1). The negative ion mode provided superior sensitivity for the majority of phenolic, organic acid, and tannin constituents, while the positive ion mode facilitated detection of alkaloid and other nitrogen-containing compounds (Supplementary Figure S1). Blank chromatograms in both negative and positive ESI modes, as well as positive-mode chromatograms for both extracts, are presented in Supplementary Figure S1.

#### 3.1.1. Tentative Identification of Compounds in *P. guajava* Extract

The aqueous extract of *P. guajava* (VJM-I-150B) yielded 11 tentatively identified compounds in negative ESI mode (Table S1). The chromatographic profile was dominated by hydrolysable tannins, flavan-3-ols, and flavonol glycosides.

Gallic acid (peak 1, RT = 1.91 min, *m*/*z* 168.0193 [M − H]^−^, mass error −6.3 ppm) was identified as a major early-eluting constituent [1,12,13]. The proanthocyanidin procyanidin B1 (peak 2, RT = 3.93 min, *m*/*z* 577.1351 [M − H]^−^, mass error 0.0 ppm) was detected as a prominent peak, alongside the flavan-3-ol monomers catechin (peak 3, RT = 4.30 min, *m*/*z* 289.0762 [M − H]^−^, mass error 17.3 ppm) and epigallocatechin (peak 4, RT = 5.39 min, *m*/*z* 305.0685 [M − H]^−^, mass error 7.9 ppm) [1,3,13].

A flavonol glycoside, kaempferol-3-O-(2,3,4-tri-*O*-acetyl-α-L-rhamnopyranoside) (peak 6, RT = 8.36 min, *m*/*z* 557.1288 [M − H]^−^, mass error −1.3 ppm), was tentatively identified, along with the methylated flavonol rhamnetin (peak 8, RT = 11.94 min, *m*/*z* 271.0603 [M − HCO_2_]^−^, mass error −3.2 ppm) [1]. The triterpenoid arjugenin (peak 7, RT = 11.67 min, *m*/*z* 549.3437 [M + HCO_2_]^−^, mass error 0.7 ppm) was also detected [1].

Three compounds (peaks 5, 9, and 10) could not be confidently assigned based on the available spectral libraries. Peak 5 (RT = 6.74 min, *m*/*z* 380.9909 [M − H]^−^) exhibited a molecular formula of C_11_H_10_O_15_ (mass error −8.4 ppm). Peaks 9 and 10 (RT = 13.81 and 14.48 min, both with *m*/*z* 491.2443 and 491.2425 [M − H]^−^, respectively) shared the same molecular formula (C_30_H_36_O_6_). Peak 11 (RT = 16.10 min, *m*/*z* 473.2318 [M − H]^−^, mass error −2.1 ppm) was tentatively identified as (1*S*,4*S*,7*R*,11*R*,12*S*)-14,16-dihydroxy-1,5,5-trimethyl-8-methylidene-12-phenyl-19-oxatetracyclo [9.8.0.0^4^,^7^.0^13^,^18^]nonadeca-13,15,17-triene-15,17-dicarbaldehyde [1,4].

#### 3.1.2. Tentative Identification of Compounds in *S. plumosum* Extract

UPLC-HRMS analysis of the aqueous extract of *S. plumosum* (VJM-I-150A) in negative ESI mode led to the tentative identification of 16 compounds, comprising organic acids, phenolic acids, flavonoids, lignans, and other specialised metabolites (Table S2). The major peaks were observed at retention times ranging from 1.35 to 14.70 min.

Citric acid (peak 1, RT = 1.35 min, *m*/*z* 191.0189 [M − H]^−^, mass error −4.1 ppm) was identified as the most polar constituent [1]. 2,4-Dihydroxybenzoic acid (peak 2, RT = 3.30 min, *m*/*z* 153.0181 [M − H]^−^, mass error −7.9 ppm) was also detected [1].

Several caffeoylquinic acid derivatives were detected. 4-Caffeoylquinic acid (peak 4, RT = 4.71 min, *m*/*z* 353.0863 [M − H]^−^, mass error −4.3 ppm) was identified, along with caffeic acid (peak 5, RT = 4.99 min, *m*/*z* 179.0339 [M − H]^−^, mass error −5.9 ppm) [1,11]. Three isomers of 1,5-dicaffeoylquinic acid (peaks 6–8, RT = 5.26, 6.79, and 7.38 min, *m*/*z* 515.1183–515.1194 [M − H]^−^, mass errors ranging from −1.4 to −0.1 ppm) were detected [1].

The flavonoid fraction included taxifolin (peak 3, RT = 4.58 min, *m*/*z* 349.0588 [M + HCO_2_]^−^, mass error 6.5 ppm), (−)-epigallocatechin (peak 9, RT = 7.63 min, *m*/*z* 261.0760 [M − HCO_2_]^−^, mass error −3.1 ppm), and chrysosplenetin (peak 10, RT = 8.21 min, *m*/*z* 329.1012 [M − HCO_2_]^−^, mass error −5.7 ppm) [1].

The isoquinoline alkaloid berberine (peak 11, RT = 10.01 min, *m/z* 395.1364 [M + CH_3_CO_2_]^−^, mass error −2.5 ppm) was detected [1,11]. Curcumin (peak 12, RT = 10.28 min, *m/z* 427.1400 [M + CH_3_CO_2_]^−^, mass error 0.3 ppm) was also tentatively identified [1]. The lignan episesartemin (peak 13, RT = 10.68 min, *m/z* 429.1559 [M − H]^−^, mass error 0.9 ppm) was detected [1]. Bergamottin (peak 16, RT = 13.22 min, *m/z* 337.1435 [M − H]^−^, mass error −3.0 ppm) was also tentatively identified [1]. Arachidonic acid methyl ester (peak 17, RT = 14.70 min, *m*/*z* 377.2714 [M + CH_3_CO_2_]^−^, mass error 4.5 ppm) was detected in the later-eluting fraction [1].

Two compounds (peaks 14 and 15) could not be confidently assigned. Peak 14 (RT = 12.16 min, *m*/*z* 311.2241 [M − H]^−^) exhibited a molecular formula of C_18_H_32_O_4_ (mass error 6.1 ppm). Peak 15 (RT = 12.61 min, *m/z* 293.2122 [M − H]^−^, mass error 1.7 ppm) was assigned a molecular formula of C_18_H_30_O_3_.

Collectively, the UPLC-PDA-HRMS analysis revealed distinct phytochemical profiles for PG and SP, with PG enriched in hydrolysable tannins and flavan-3-ols, while SP exhibited greater diversity in caffeoylquinic acid derivatives, alkaloids, and lignans. These chemical differences provide a foundation for interpreting the observed biological activities and synergistic interactions presented in subsequent sections.

### 3.2. Cell Viability in HepG2 Human Hepatoma Cells

**Figure 2:**
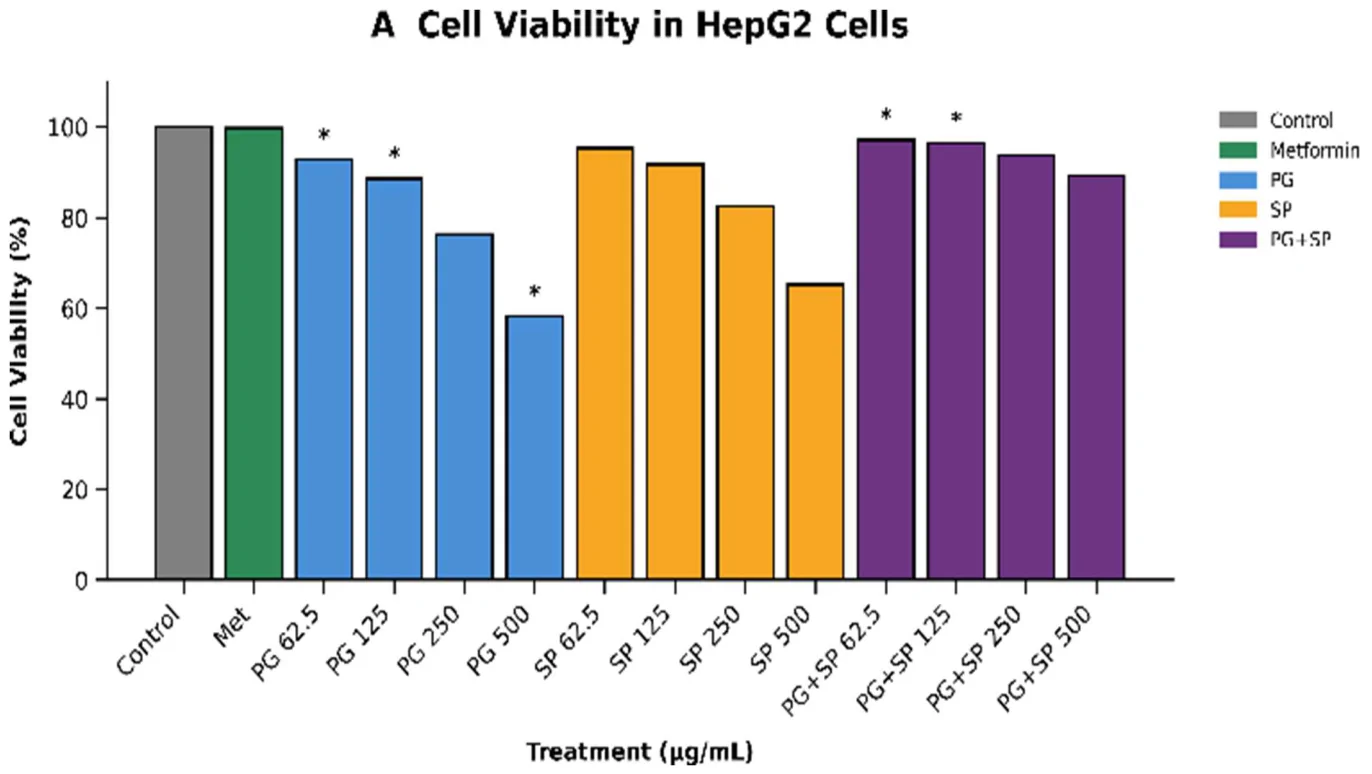

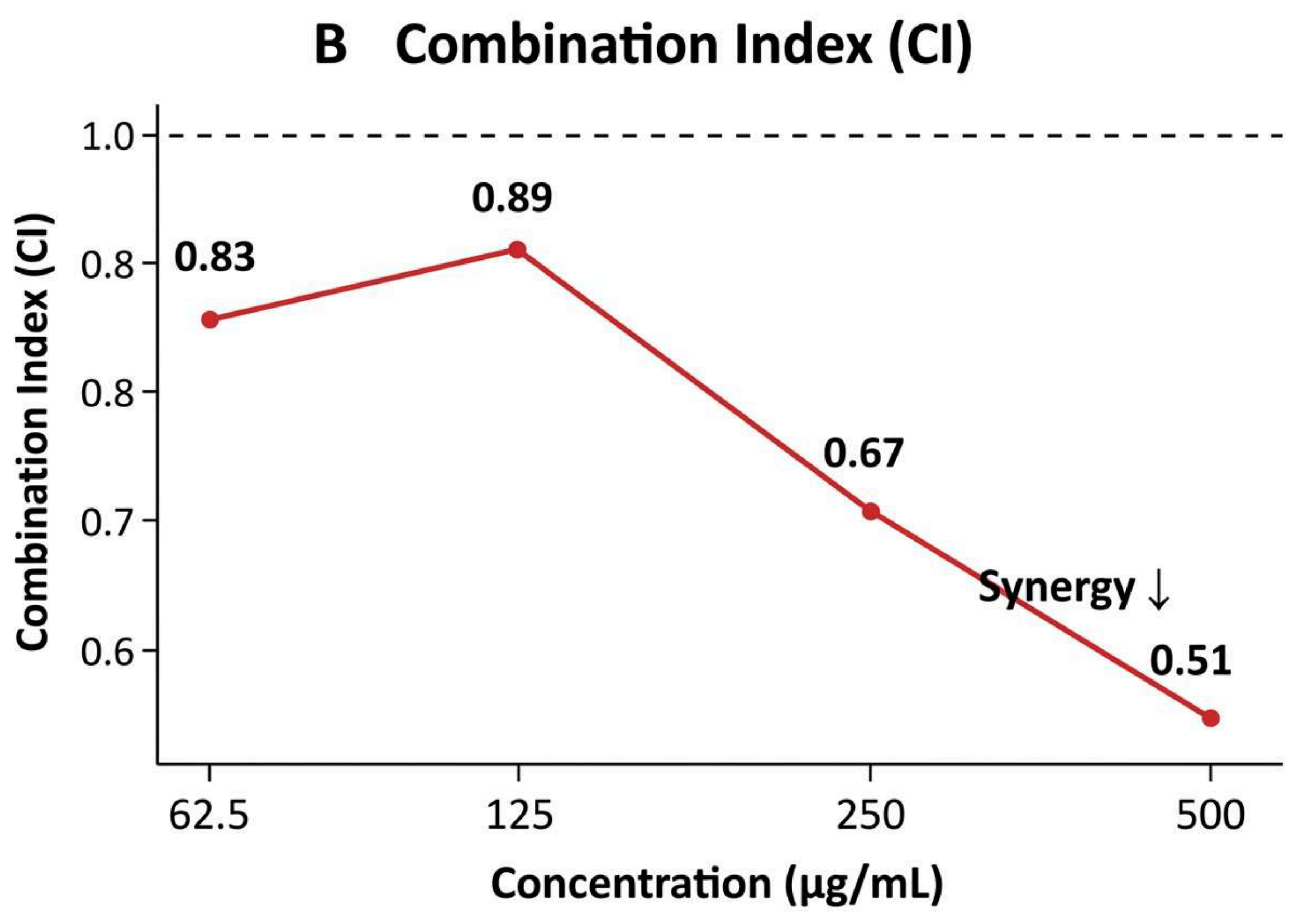
Effects of *Psidium guajava* (PG), *Seriphium plumosum* (SP), and their 1:1 combination on HepG2 cell viability following 24 h exposure. (**A**) Cell viability (%) following treatment with PG, SP, and PG+SP at concentrations of 62.5, 125, 250, and 500 µg/mL. Data are presented as mean ± SD from three independent experiments (*n* = 3). PG produced a concentration-dependent reduction in viability, whereas SP exhibited comparatively low cytotoxicity. The PG+SP combination maintained higher viability than extract alone across all concentrations tested. Statistical significance relative to the untreated control is indicated as * *p* < 0.05. (**B**) Bliss Independence and Combination Index (CI) analyses illustrating concentration-dependent interactions between *P. guajava* (PG) and *S. plumosum* (SP). Lower concentrations demonstrated predominantly additive interactions, whereas higher concentrations showed synergistic cytoprotective effects. The ↓ symbol indicates increasing synergy with rising concentration. The dashed line at CI = 1.0 represents the threshold for additivity. CI < 1 indicates synergy, CI = 1 additivity, and CI > 1 antagonism.

The cytotoxic effects of *Psidium guajava* (PG), *Seriphium plumosum* (SP), and their 1:1 combination were evaluated in HepG2 human hepatoma cells following 24 h exposure (Figure 2A). PG exhibited a concentration-dependent reduction in cell viability, decreasing from 92.8 ± 1.8% at 62.5 µg/mL to 58.2 ± 4.5% at 500 µg/mL (Figure 2A). Statistically significant reductions in viability were observed at 62.5 µg/mL (*p* = 0.008), 125 µg/mL (*p* = 0.042), and 500 µg/mL (*p* = 0.021), whereas the effect at 250 µg/mL was not statistically significant (*p* = 0.089), indicating a non-linear dose–response pattern. In contrast, SP demonstrated comparatively low cytotoxicity across the tested concentration range. Cell viability ranged from 95.4 ± 1.2% to 65.3 ± 3.6%, with no statistically significant differences relative to the untreated control (*p* > 0.05). These findings suggest that SP is less cytotoxic than PG under the experimental conditions, with a wider tolerance profile in HepG2 cells. The 1:1 PG+SP combination consistently maintained higher cell viability than extract alone across all concentrations tested. Viability remained above 89% at all doses, with values of 97.2 ± 0.8%, 96.5 ± 1.1%, 93.8 ± 1.4%, and 89.4 ± 1.8% at 62.5, 125, 250, and 500 µg/mL, respectively. Statistically significant differences relative to the untreated control were observed at 62.5 and 125 µg/mL (*p* = 0.001 and *p* = 0.002), while no significant differences were detected at higher concentrations (*p* = 0.089 and *p* = 0.058).

Interaction analysis using Bliss independence and the Combination Index (CI) revealed concentration-dependent effects of the PG+SP combination. At lower concentrations (62.5 and 125 µg/mL), Bliss scores (+3.1 and +6.3) indicated additive interactions, while higher concentrations (250 and 500 µg/mL) showed modest synergistic effects (+14.3 and +27.6). This pattern was corroborated by CI analysis, where values decreased from 0.83 and 0.89 (additive range) to 0.67 and 0.51, confirming mild-to-moderate synergistic cytoprotective interactions at higher concentrations. iMetformin, used as a positive control, maintained cell viability at 99.8 ± 2.4% (*p* = 0.989), confirming the absence of cytotoxic effects under the experimental conditions.

### 3.3. Glucose Uptake in HepG2 Cells

**Figure 3:**
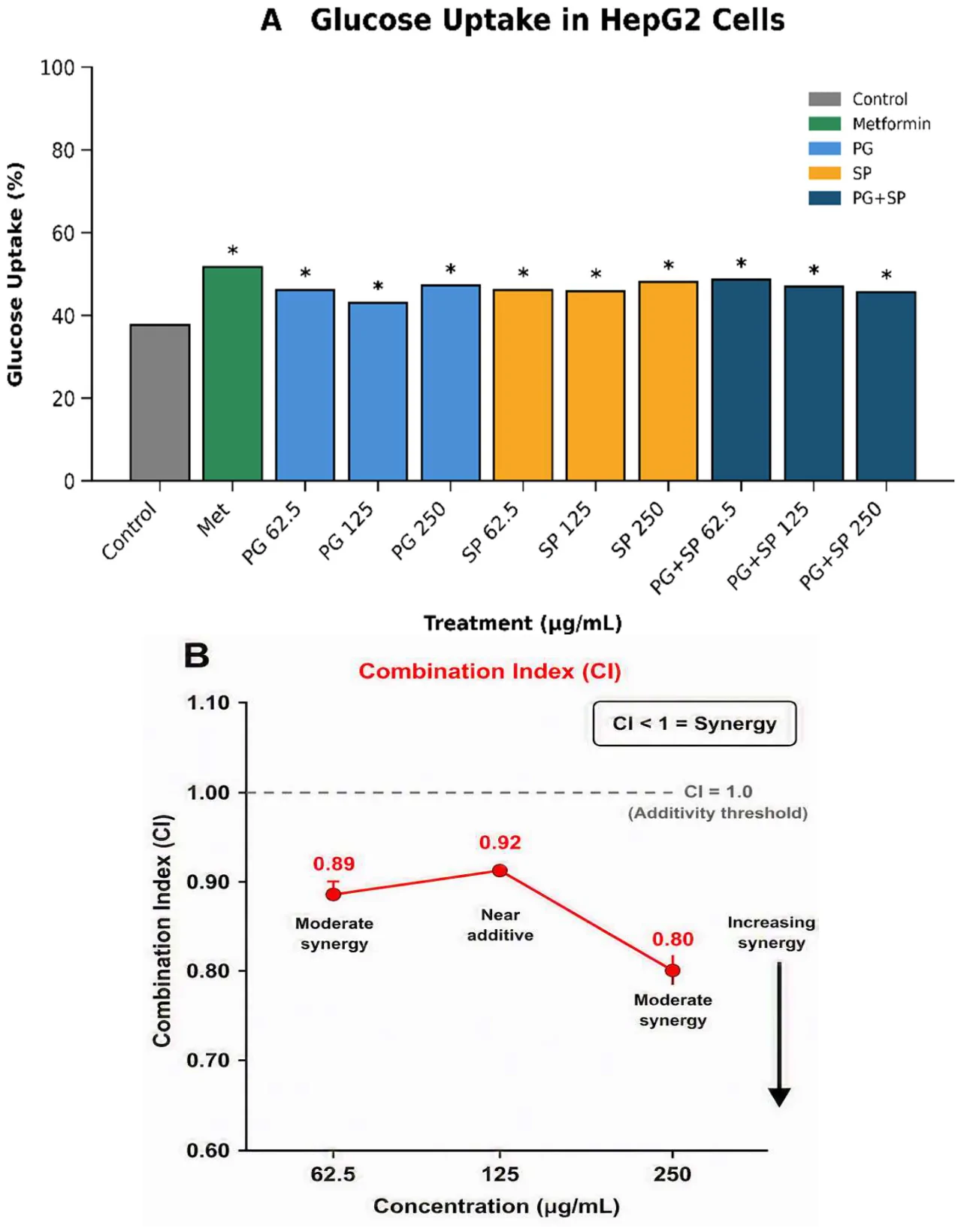
Effects of *Psidium guajava* (PG), *Seriphium plumosum* (SP), and their 1:1 combination on glucose uptake in HepG2 human hepatoma cells. (**A**) Glucose uptake (%) following 24 h treatment with PG, SP, and PG+SP at concentrations of 62.5, 125, and 250 µg/mL. Data are presented as mean ± SEM from three independent experiments performed in triplicate (*n* = 2). All treatments significantly increased glucose uptake relative to the untreated control (*p* < 0.05), with SP producing the highest single-agent response at 250 µg/mL (48.01 ± 0.41%) and the PG+SP combination achieving 48.59 ± 0.28% glucose uptake at 62.5 + 62.5 µg/mL, corresponding to approximately 94% of the metformin response. Asterisks (*) indicate statistically significant differences (*p* < 0.05) compared with the untreated control. (**B**) Combination Index (CI) analysis of PG+SP interactions. The ↓ symbol indicates increasing synergy with rising concentration. The dashed line represents the additivity threshold (CI = 1), with values below 1 indicating increasing synergy. CI values ranging from 0.80 to 0.92 indicate concentration-dependent interactions varying from near-additive to moderately synergistic effects on hepatic glucose uptake.

Both *Psidium guajava* (PG) and *Seriphium plumosum* (SP) individually enhanced glucose uptake in HepG2 cells relative to the untreated control (37.71 ± 0.15%) across all tested concentrations (Figure 3A). SP at 250 µg/mL produced the highest single-agent response (48.01 ± 0.41%, *p* = 0.0054), while PG also induced a consistent increase in glucose uptake across concentrations, indicating a moderate glucose-lowering potential in hepatic cells.

The 1:1 PG+SP combination further enhanced glucose uptake, with the greatest effect observed at 62.5 µg/mL (48.59 ± 0.28%, *p* = 0.0041). This response corresponded to 94.2% of the metformin positive control (51.61 ± 0.18% at 160 µg/mL), indicating a comparable functional effect at lower concentrations. At higher combination doses, glucose uptake remained elevated but did not exceed the peak response observed at the lowest combination concentration, suggesting a non-linear dose–response relationship.

Interaction analysis using the Combination Index (CI) indicated concentration-dependent effects ranging from mild synergy to near-additive interactions. CI values of 0.89, 0.92, and 0.80 were observed at 62.5, 125, and 250 µg/mL, respectively, with the strongest interaction occurring at 250 µg/mL. Overall, these findings suggest that PG and SP interact to enhance hepatic glucose uptake primarily through complementary rather than strictly dose-dependent synergistic mechanisms. Metformin consistently produced a higher glucose uptake response than individual extracts and served as a pharmacological benchmark for comparison. The CI values of 0.51–0.89 indicate mild-to-moderate synergy, with the degree of interaction dependent on the concentration and analytical method employed.

### 3.4. GLUT2 Protein Expression in HepG2 Cells

**Figure 4:**
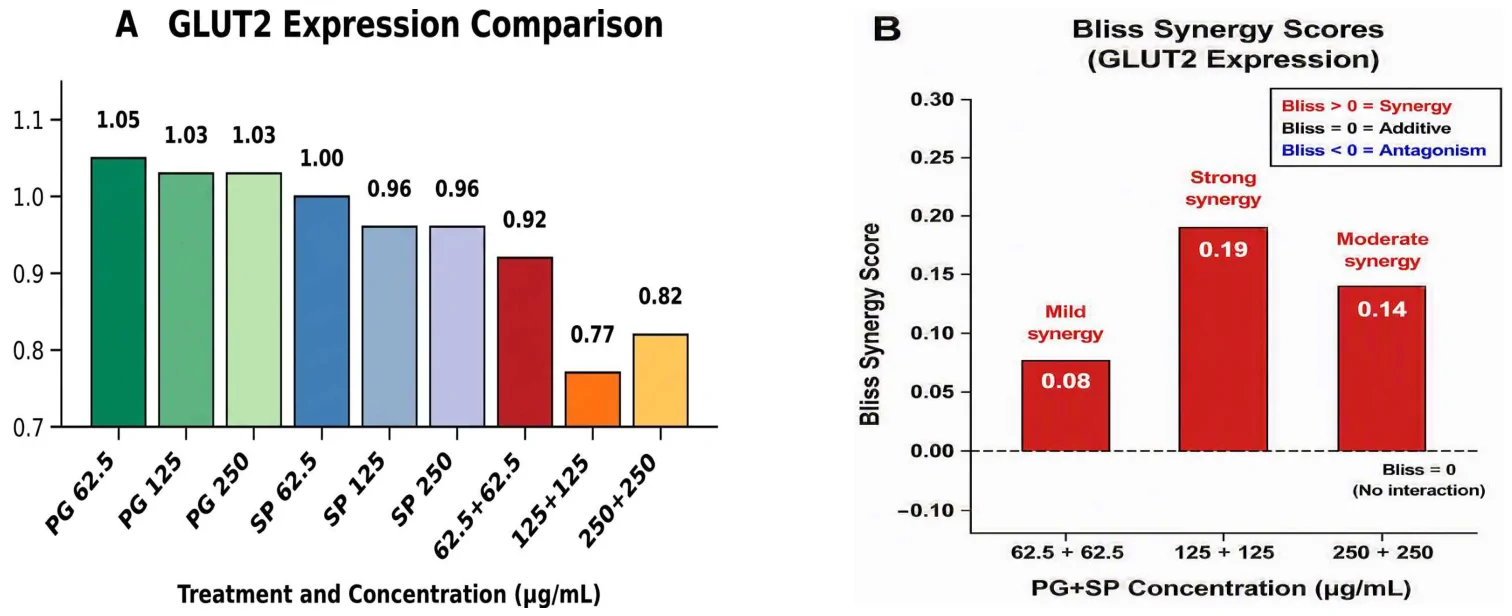
Effects of *Psidium guajava* (PG), *Seriphium plumosum* (SP), and their 1:1 combination on GLUT2 protein expression in HepG2 human hepatoma cells. (**A**) GLUT2 protein expression expressed as fold change relative to the untreated control following 24 h treatment with PG, SP, and PG+SP at concentrations of 62.5, 125, and 250 µg/mL. Data are presented as mean ± SD from three independent experiments (*n* = 3). PG and SP alone produced minimal effects on GLUT2 expression, whereas the PG+SP combination induced concentration-dependent downregulation, with the greatest reduction observed at 125 + 125 µg/mL (0.77 ± 0.14-fold). At this concentration, the combination produced greater GLUT2 suppression than metformin (0.84 ± 0.29-fold). (**B**) Bliss Independence synergy analysis for GLUT2 modulation. The dashed line indicates the synergy threshold (Bliss score = 0.10). Positive Bliss scores across all combination concentrations indicate additive-to-synergistic interactions, with the strongest interaction observed at 125 + 125 µg/mL (Bliss score = +0.19).

GLUT2 protein expression was assessed in HepG2 cells following treatment with *Psidium guajava* (PG), *Seriphium plumosum* (SP), and their 1:1 combination, and expressed as fold change relative to the untreated control (Figure 4A). Overall, neither PG nor SP alone produced substantial changes in GLUT2 expression across the tested concentration range, with fold changes remaining close to baseline (0.96–1.05), indicating minimal effect on basal GLUT2 protein levels under the experimental conditions.

In contrast, the PG+SP combination induced a concentration-dependent modulation of GLUT2 expression. A modest reduction was observed at 62.5 µg/mL (0.92 ± 0.09), while a more pronounced and statistically significant decrease occurred at 125 µg/mL (0.77 ± 0.14, *p* < 0.0001). A partial recovery was observed at 250 µg/mL (0.82 ± 0.37, *p* < 0.0001), although GLUT2 levels remained below those of the untreated control. At 125 µg/mL, GLUT2 expression was also lower than that observed with metformin (0.84 ± 0.29), suggesting a comparatively stronger suppressive effect at this concentration.

CI values of 0.89, 0.92, and 0.80 were observed at 62.5, 125, and 250 µg/mL, respectively, indicating mild-to-moderate synergy with the most notable interaction observed at 250 µg/mL. Overall, these findings suggest that PG and SP interact to enhance hepatic glucose uptake primarily through complementary rather than potent synergistic mechanisms. Although the magnitude of change was moderate, the consistent direction of effect across synergy models supports a biologically meaningful interaction between PG and SP in regulating hepatic glucose transport.

Given that GLUT2 functions as the primary bidirectional glucose transporter in hepatocytes, these findings may indicate a compensatory regulatory response in which enhanced glucose uptake is accompanied by downregulation of GLUT2 expression. This pattern is consistent with glucose-sensing feedback mechanisms that act to limit excessive intracellular glucose accumulation and maintain hepatic metabolic homeostasis.

### 3.5. Loewe Additivity and ZIP Synergy Analysis in HepG2 Cells

**Figure 5:**
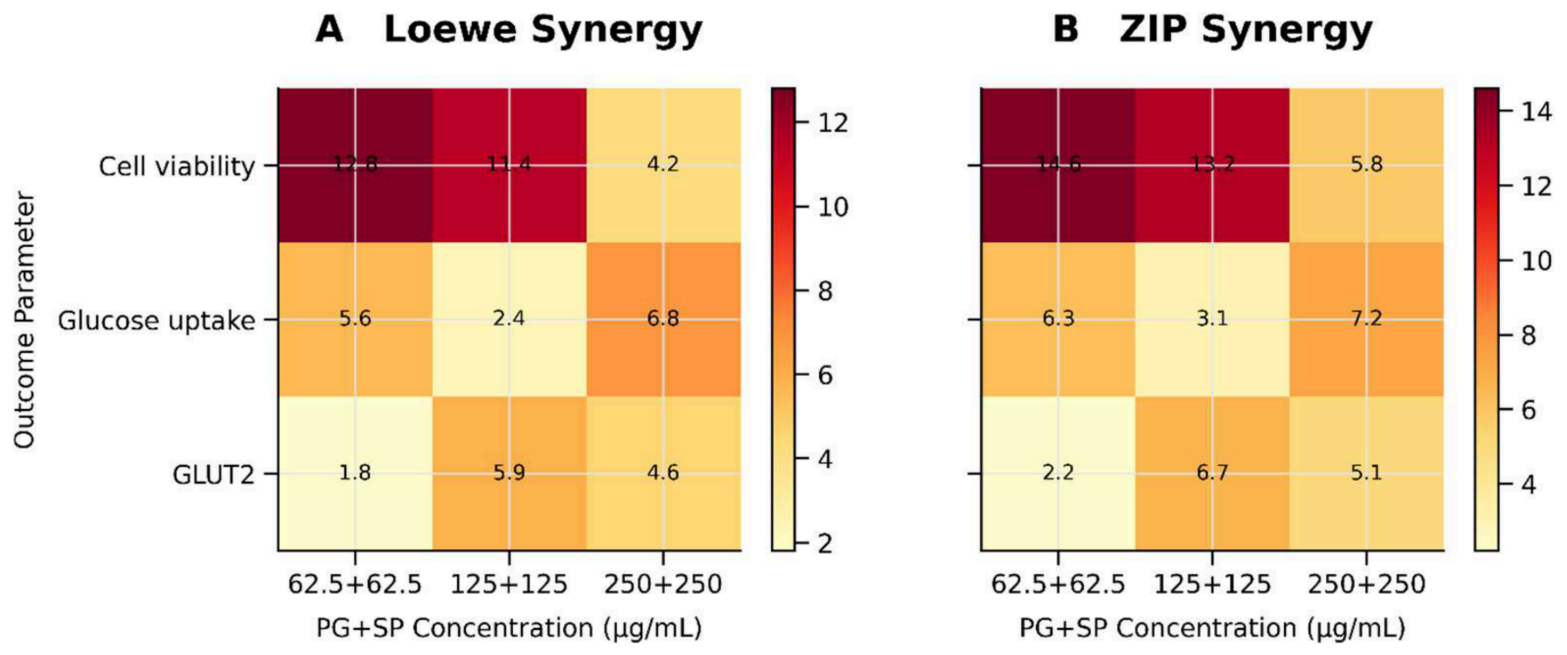
Heatmap representation of Loewe and ZIP synergy scores for the combination of *Psidium guajava* (PG) and *Seriphium plumosum* (SP) in HepG2 human hepatoma cells. Heatmaps illustrate interaction patterns across cell viability, glucose uptake, and GLUT2 protein expression endpoints following treatment with *Psidium guajava* (PG) and *Seriphium plumosum* (SP) combinations. Rows represent biological endpoints, and columns represent combination concentrations (62.5 + 62.5, 125 + 125, and 250 + 250 µg/mL). Values within each cell correspond to the calculated Loewe and ZIP synergy scores. Colour intensity reflects the magnitude of the interaction, with darker colours indicating stronger synergistic effects. Cell viability demonstrated the strongest interaction at lower concentrations according to both models, whereas glucose uptake and GLUT2 modulation exhibited predominantly mild-to-moderate synergistic effects across concentrations. Positive scores indicate synergy, values near zero indicate additivity, and negative values indicate antagonism.

To further validate the interaction between *Psidium guajava* (PG) and *Seriphium plumosum* (SP), synergy was assessed using the Loewe additivity and Zero Interaction Potency (ZIP) models in addition to Bliss and Combination Index (CI) analyses (Figure 5). This multi-model approach was employed to account for the different mathematical assumptions underlying synergy quantification and to improve the robustness of interaction classification. For cell viability, Loewe and ZIP models indicated strongest synergy at 62.5 and 125 µg/mL, with scores exceeding +10, while Bliss and CI showed the opposite trend—weaker synergy at low concentrations and stronger synergy at 250–500 µg/mL. This divergence likely reflects differing sensitivity of the two model families to the curvature of the dose–response relationship at the extremes of the tested range, rather than a shared concentration-dependent pattern, and should be interpreted with appropriate caution rather than treated as convergent evidence.

For glucose uptake, both the Loewe and ZIP models indicated mild to moderate synergy at 62.5 and 250 µg/mL, while the 125 µg/mL combination exhibited near-additive effects. These findings suggest that the enhancement of hepatic glucose uptake is partially concentration-dependent and may not follow a strictly linear synergistic pattern across all doses.

For GLUT2 protein expression, both models demonstrated low-to-moderate synergistic modulation, with stronger effects observed at 125 and 250 µg/mL. Although the magnitude of interaction was smaller compared to cell viability outcomes, the direction of effect remained consistent across models, indicating reproducible combinatorial modulation of hepatic glucose transport regulation. Importantly, across all evaluated endpoints (cell viability, glucose uptake, and GLUT2 expression), at least three independent synergy models (Bliss, CI, Loewe, and ZIP) showed concordant interaction patterns, supporting the robustness of the observed synergistic effects. This convergence across mechanistically distinct models strengthens confidence that the interactions between PG and SP are not artefacts of a single analytical framework but represent a consistent biological phenomenon. Overall, the PG+SP combination exhibited concentration- and endpoint-dependent interactions ranging from additivity to synergy, highlighting complementary rather than uniformly synergistic activity.

The divergence between model families observed in this study—where Loewe and ZIP indicated stronger synergy at lower concentrations while Bliss and CI showed stronger synergy at higher concentrations—likely reflects differences in sensitivity to dose–response curvature rather than true biological inconsistency [27,28]. This highlights that synergy is context-dependent and reinforces the importance of multi-model approaches in polyherbal research. Because no individual synergy model is universally accepted as definitive, convergence across multiple independent analytical frameworks was considered stronger evidence of biological interaction than reliance on a single method.

### 3.6. Cell Viability in C2C12 Myoblasts

**Figure 6:**
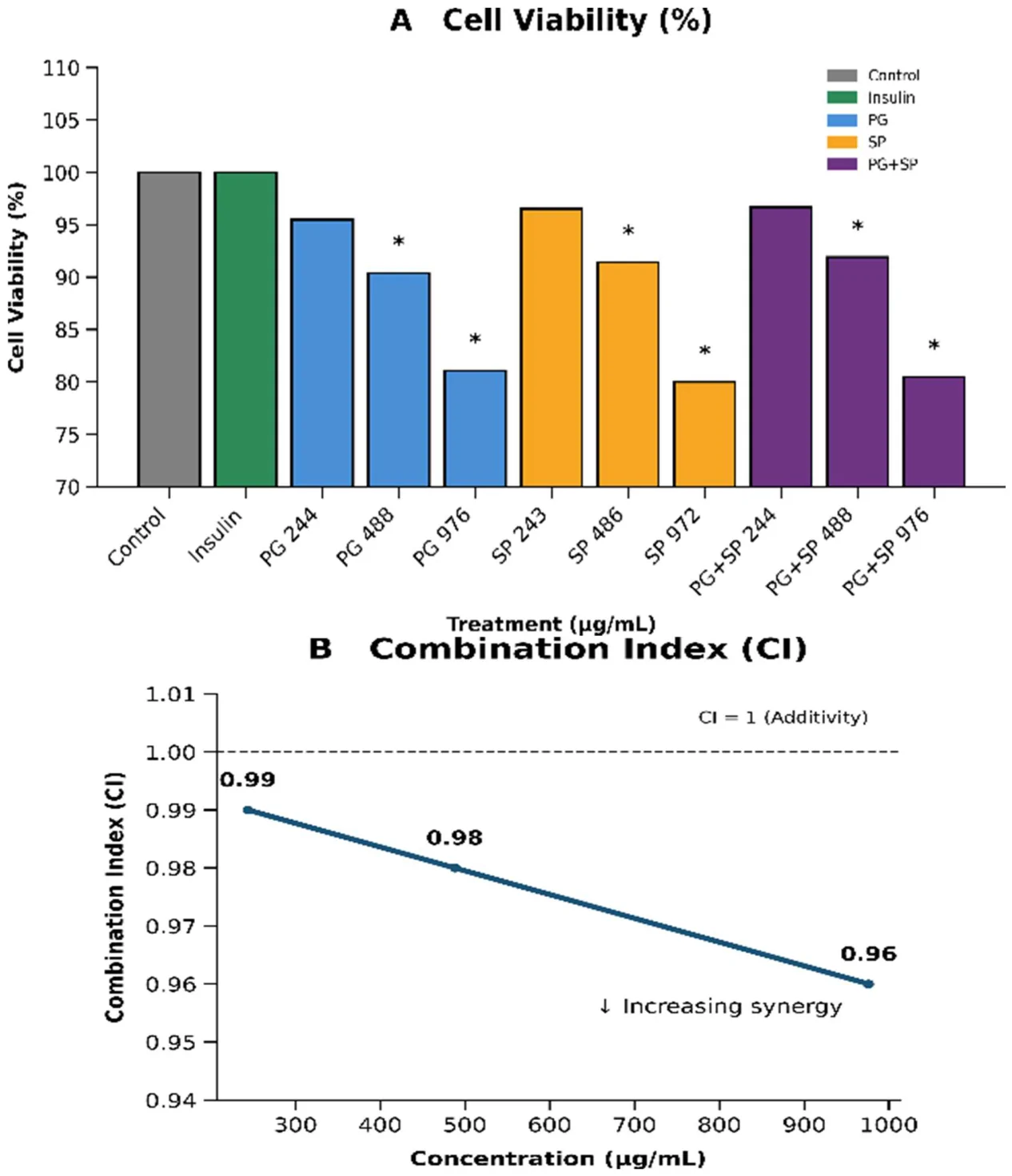
Effects of *Psidium guajava* (PG), *Seriphium plumosum* (SP), and their 1:1 combination on cell viability in C2C12 mouse skeletal muscle myoblasts. (**A**) Cell viability (%) following 24-h exposure to PG, SP, and PG+SP (1:1 combination) at concentrations of 244–976 µg/mL, alongside untreated control and insulin (100 nM) as a positive control. Values represent mean responses (*n* = 3). Statistical significance relative to untreated control is indicated as * *p* < 0.05. (**B**) Combination Index (CI) values for PG+SP interactions across concentrations. The ↓ symbol indicates increasing synergy with rising concentration. The dashed line represents the additivity threshold (CI = 1), and values close to 1 indicate additive interactions, while decreasing CI values reflect increasing synergistic interaction.

Prior to mechanistic assays, the cytotoxic effects of Psidium guajava (PG), Seriphium plumosum (SP), and their 1:1 combination were evaluated in C2C12 mouse skeletal muscle myoblasts over a concentration range of 100–3000 µg/mL (Figure 6A). PG induced a concentration-dependent reduction in cell viability, with significant decreases observed at 488 µg/mL (90.4 ± 4.0%, *p* = 0.042) and 976 µg/mL (81.1 ± 5.0%, *p* = 0.008). Similarly, SP exhibited significant reductions at 486 µg/mL (91.4 ± 3.8%, *p* = 0.038) and 972 µg/mL (80.0 ± 5.2%, *p* = 0.006), confirming mild cytotoxicity at higher concentrations for both extracts. The 1:1 PG+SP combination maintained cell viability close to the predicted additive response across all tested concentrations, with values of 96.7 ± 3.3%, 91.9 ± 3.9%, and 80.5 ± 5.1% at 244/243, 488/486, and 976/972 µg/mL, respectively. Although a slight reduction in viability was observed at higher concentrations, all values remained at or above 80%, confirming suitability for downstream metabolic assays.

Interaction analysis using Bliss independence and the Combination Index (CI) indicated predominantly additive interactions for cytotoxicity in C2C12 cells (Figure 6B). Bliss scores ranged from +0.02 to +0.09, remaining within the additive interaction threshold, while CI values ranged from 0.96 to 0.99, indicating near-additive to weakly synergistic effects. Overall, these findings demonstrate that the PG+SP combination does not induce enhanced cytotoxicity in skeletal muscle cells and remains compatible with physiological metabolic assessment conditions. Based on these cytotoxicity data, concentrations of 244, 488, and 976 µg/mL for PG and 243, 486, and 972 µg/mL for SP, and their corresponding 1:1 combination, were selected for subsequent mechanistic experiments.

### 3.7. Glucose Uptake in C2C12 Myotubes

**Figure 7:**
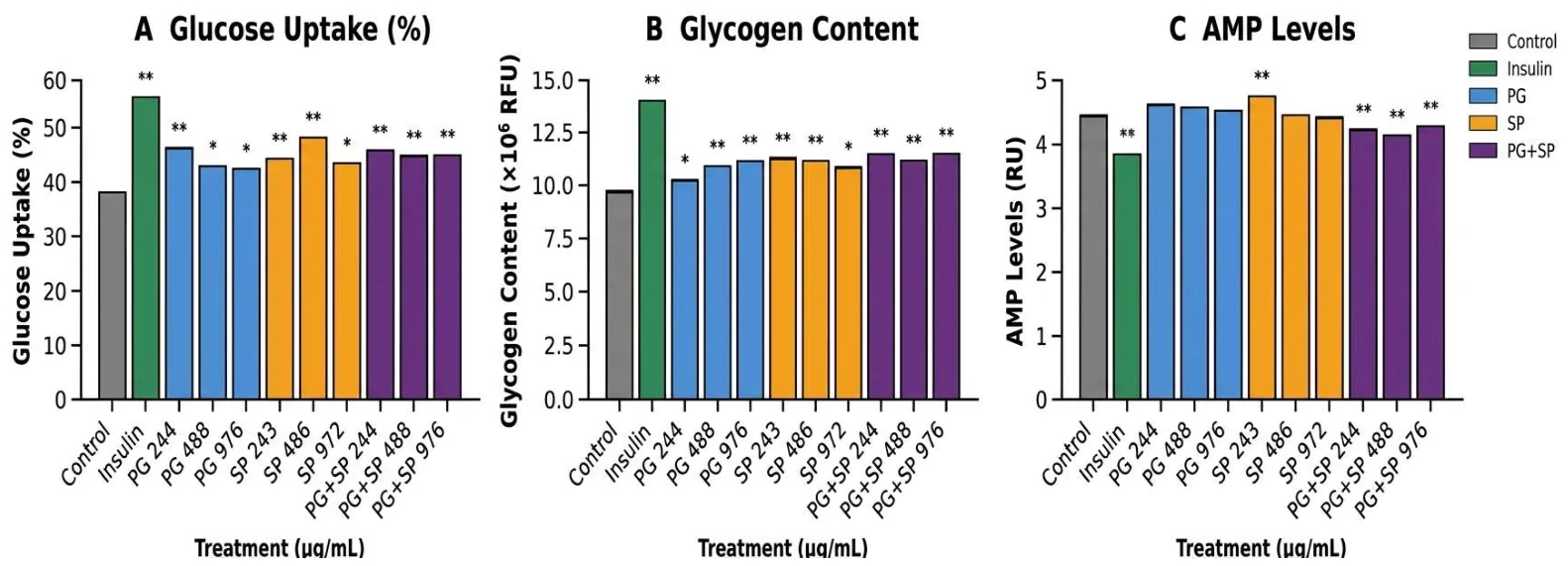
Effects of *Psidium guajava* (PG), *Seriphium plumosum* (SP), and their 1:1 combination on glucose metabolism and energy regulation in differentiated C2C12 myotubes. (**A**) Glucose uptake (%) following treatment with PG, SP, and PG+SP at the indicated concentrations. (**B**) Glycogen content (×10^6^ RFU) as a measure of intracellular glucose storage. (**C**) AMP levels (RU) as an indicator of cellular energy status. Insulin (100 nM) served as the positive control. Data are presented as mean ± SEM from three independent experiments (*n* = 3). Statistical significance relative to the untreated control is indicated as * *p* < 0.05, ** *p* < 0.01. RFU, relative fluorescence units; RU, relative units.

The effects of PG, SP, and their 1:1 combination on glucose uptake were evaluated in differentiated C2C12 myotubes (Figure 7A). All treatments significantly increased glucose uptake relative to the untreated control (38.05 ± 0.15%). Among the individual treatments, SP at 486 µg/mL produced the highest glucose uptake response (48.09 ± 0.22%, *p* < 0.01), whereas PG increased glucose uptake across all tested concentrations, with values ranging from 42.42 ± 0.30% to 46.14 ± 0.32%. The PG+SP combination also enhanced glucose uptake, producing responses of 45.73 ± 0.28%, 44.74 ± 0.26%, and 44.90 ± 0.31% at 244 + 243, 488 + 486, and 976 + 972 µg/mL, respectively. Insulin treatment resulted in the greatest stimulation of glucose uptake (55.47 ± 0.18%, *p* < 0.01) and served as the positive control.

### 3.8. Glycogen Content in C2C12 Myotubes

Intracellular glycogen content was measured following treatment with PG, SP, and their 1:1 combination (Figure 7B). Relative to the untreated control (9.36 ± 0.12 × 10^6^ RFU), all treatments significantly increased glycogen accumulation. PG treatment increased glycogen content to values ranging from 9.85 ± 0.08 × 10^6^ RFU to 10.70 ± 0.09 × 10^6^ RFU, whereas SP produced values between 10.40 ± 0.10 × 10^6^ RFU and 10.80 ± 0.11 × 10^6^ RFU. The PG+SP combination resulted in the highest glycogen accumulation, reaching 11.00 ± 0.09 × 10^6^ RFU at 244 + 243 µg/mL and 11.00 ± 0.12 × 10^6^ RFU at 976 + 972 µg/mL (*p* < 0.01). Insulin produced the strongest glycogen response, increasing glycogen content to 13.40 ± 0.15 × 10^6^ RFU (*p* < 0.01).

### 3.9. AMP Levels in C2C12 Myotubes

AMP levels were quantified as an indicator of cellular energy status following treatment with PG, SP, and their 1:1 combination (Figure 7C). The untreated control exhibited an AMP level of 3.87 ± 0.05 RU. PG treatment did not significantly alter AMP levels, with values ranging from 3.94 ± 0.06 RU to 4.02 ± 0.04 RU (*p* > 0.05). In contrast, SP at 243 µg/mL significantly increased AMP levels to 4.14 ± 0.05 RU (*p* < 0.01), whereas no significant differences were observed at the higher SP concentrations. The PG+SP combination reduced AMP levels to 3.68 ± 0.06 RU, 3.61 ± 0.05 RU, and 3.73 ± 0.07 RU at 244 + 243, 488 + 486, and 976 + 972 µg/mL, respectively. Insulin treatment produced the lowest AMP level (3.35 ± 0.04 RU, *p* < 0.01).

### 3.10. Bliss Independence Synergy Analysis in C2C12 Myoblasts

**Figure 8:**
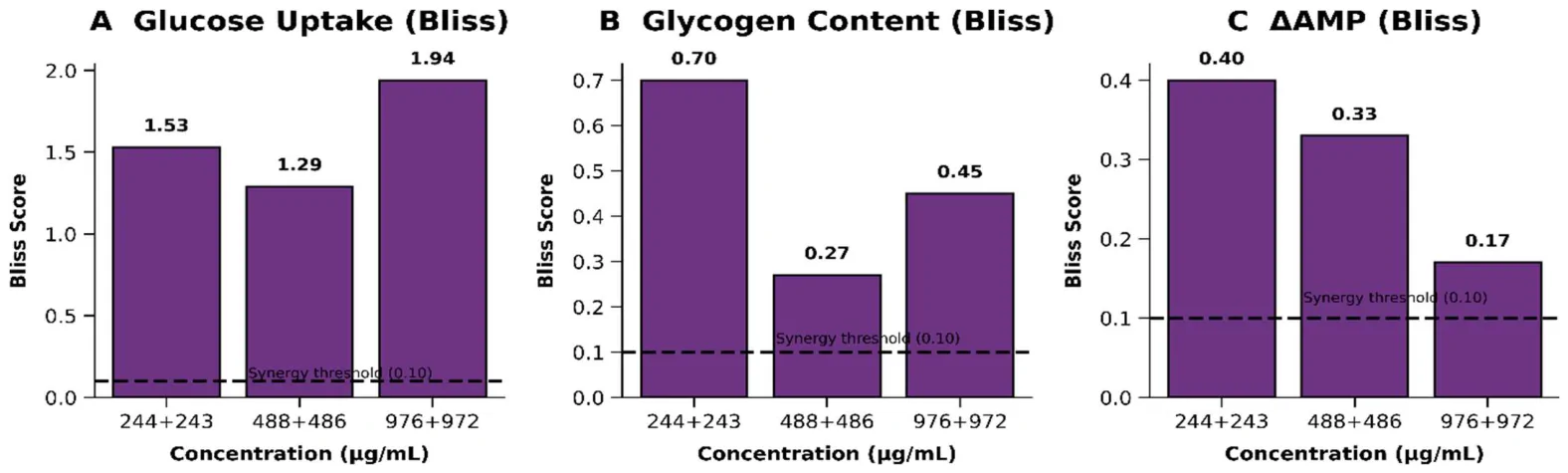
Effects of *Psidium guajava* (PG), *Seriphium plumosum* (SP), and their 1:1 combination on glucose metabolism and energy regulation in differentiated C2C12 mouse skeletal muscle myotubes. (**A**) Glucose uptake, (**B**) glycogen content, and (**C**) ΔAMP levels across PG+SP concentration combinations. Positive Bliss scores indicate synergistic interactions relative to predicted additive effects. Data represent mean values (*n* = 3).

Bliss Independence modelling was used to quantify interaction effects between PG and SP across glucose uptake, glycogen storage, and cellular energy status (Figure 8A–C, Table 1). For glucose uptake, all combination doses exhibited positive Bliss scores (+1.29 to +1.94), confirming synergistic enhancement beyond predicted additive effects. The strongest interaction was observed at 976 µg/mL (+1.94). For glycogen content, the combination produced predominantly synergistic effects at low and high concentrations (+0.70 and +0.45), with an additive interaction at the intermediate dose (+0.27), indicating a non-linear concentration dependence. For AMP levels, data were normalised as ΔAMP (Control − AMP) to align directional interpretation, where higher values reflect improved cellular energy status. Following transformation, all combination treatments produced positive Bliss scores (+0.17 to +0.40), indicating synergistic improvement in energy homeostasis. The strongest effect occurred at 244 µg/mL (+0.40). Overall, Bliss analysis indicates that PG and SP interact in skeletal muscle cells, with additive-to-synergistic effects observed across endpoints, particularly in enhancing glucose uptake and restoring cellular energy balance. The magnitude of synergy was modest, with Bliss scores ranging from +0.17 to +1.94 depending on the endpoint and concentration.

### 3.11. Loewe and ZIP Synergy Analysis in C2C12 Myoblasts

**Figure 9:**
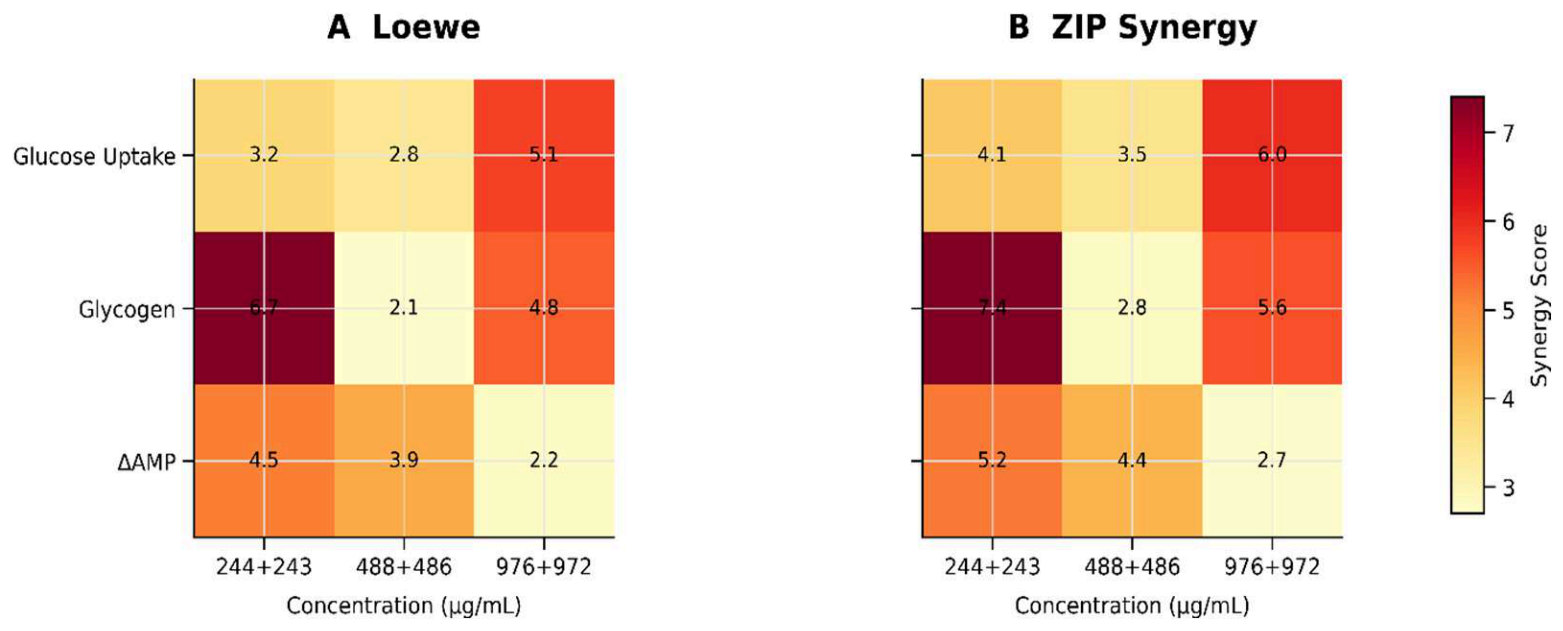
Heatmap representation of Loewe and ZIP synergy scores for the combination of *Psidium guajava* (PG) and *Seriphium plumosum* (SP) in differentiated C2C12 mouse skeletal muscle myotube (**A**) Loewe additivity scores and (**B**) ZIP synergy scores for glucose uptake, glycogen content, and ΔAMP responses across PG+SP concentration combinations (244 + 243, 488 + 486, and 976 + 972 µg/mL). Rows represent outcome parameters, while columns represent concentration combinations. Colour intensity (yellow to red gradient) corresponds to the magnitude of the synergy score, with darker colours indicating stronger synergistic interactions. A common colour scale (legend) is shown on the far right and applies to both panels. Values displayed within each cell represent the corresponding Loewe or ZIP scores. Positive scores indicate synergistic interactions, while values closer to zero indicate additive effects.

Loewe additivity and ZIP synergy analyses were also performed to confirm the Bliss Independence findings (Figure 9A,B; Table 2). For glucose uptake, both models indicated mild to moderate synergy across all concentrations, with the strongest synergy observed at 976 µg/mL (Loewe: +5.1; ZIP: +6.0). For glycogen content, moderate synergy was observed at 244 and 976 µg/mL, with additive effects at 488 µg/mL. For ΔAMP levels, moderate synergy was observed at 244 µg/mL (Loewe: +4.5; ZIP: +5.2), with mild synergy at 488 µg/mL and additive-to-mild synergy at 976 µg/mL.

## 4. Discussion

This study provides the first mechanistic evaluation of the combined effects of *Psidium guajava* (PG) and *Seriphium plumosum* (SP) on hepatic and skeletal muscle glucose metabolism using complementary live-cell models. Although both species have individually demonstrated antidiabetic activity, their interactive effects have not previously been examined. By integrating functional metabolic assays with four independent synergy models (Bliss, Combination Index (CI), Loewe, and ZIP), this study advances current understanding of African polyherbal therapies beyond descriptive efficacy and offers a more rigorous framework for evaluating botanical combinations [27,28].

### 4.1. Hepatic Cytotoxicity and Viability Modulation

The distinct cytotoxicity profiles of PG and SP highlight important differences in hepatic safety. SP maintained relatively high cell viability across concentrations, whereas PG exhibited concentration-dependent cytotoxicity, suggesting that its biological activity may be associated with cellular stress at higher doses. PG exhibited a clear concentration-dependent decline in HepG2 cell viability, decreasing from 92.8% at 62.5 µg/mL to 58.2% at 500 µg/mL [36]. Importantly, the combination consistently preserved higher viability relative to PG alone, indicating that SP may mitigate PG-associated cytotoxic effects. This suggests a protective interaction rather than simple additivity, likely arising from complementary effects of bioactive constituents on cellular survival pathways. In the context of T2DM, where hepatocyte dysfunction contributes to insulin resistance and NAFLD progression, such cytoprotective activity alongside metabolic enhancement may represent a biologically advantageous property [7,8].

### 4.2. Hepatic Glucose Uptake and GLUT2 Regulation

Both extracts enhanced glucose uptake, but their combination produced more consistent responses across concentrations, indicating functional complementarity rather than redundancy. This implies that distinct phytochemicals within PG and SP may engage different regulatory mechanisms governing hepatic glucose metabolism. The UPLC-PDA-HRMS analysis revealed distinct phytochemical profiles for PG and SP (Section 3.1; Supplementary Tables S1 and S2). PG was enriched in hydrolysable tannins (gallic acid, procyanidin B1) and flavan-3-ols (catechin, epigallocatechin) [50], consistent with earlier reports of complex guava tannins such as guajavins, psidinins, and psiguavin isolated from guava bark. In addition, guava leaves have yielded structurally novel meroterpenoids like guajadial [51], underscoring the phytochemical diversity of PG. SP contained caffeoylquinic acid derivatives, berberine, and curcumin, compounds with documented glucose-lowering properties [50,51,52,53,54,55], including antiglycation and hypoglycaemic effects demonstrated in related African medicinal plant extracts [55]. These chemical differences may contribute to their complementary effects on hepatic glucose metabolism. The concurrent enhancement of glucose uptake and suppression of GLUT2 expression suggests coordinated regulation of glucose handling. This pattern may improve intracellular glucose retention while limiting excessive bidirectional flux, thereby reducing futile glucose cycling and potential glucotoxicity under hyperglycaemic conditions [42]. Given that GLUT2 is primarily regulated by intracellular glucose availability and transcriptional factors such as HNF1α, rather than directly by insulin signalling, these findings indicate a mechanism that may differ from conventional insulin-sensitising therapies [39,40,41,42]. Previous studies on single botanical extracts have generally reported either enhanced glucose uptake or modulation of GLUT2 expression independently [30,31]. In contrast, the present study demonstrates that a polyherbal combination can simultaneously influence both processes, suggesting that interactions between phytochemical constituents may produce emergent effects not observed with individual extracts. However, these interpretations remain inferential and require validation through transcriptional and signalling pathway analyses.

### 4.3. Skeletal Muscle Glucose Metabolism and Energy Regulation

In skeletal muscle cells, the combined extracts produced coordinated effects across glucose uptake, glycogen storage, and cellular energy balance. The presence of berberine and curcumin in SP, and catechin and epigallocatechin in PG (Section 3.1; Supplementary Tables S1 and S2), may underlie the observed effects on glucose uptake and glycogen storage in C2C12 myotubes, as these compounds have been reported to modulate AMPK and insulin signalling pathways [21,22,53]. Increased glucose uptake was accompanied by enhanced glycogen accumulation, indicating effective coupling between glucose transport and storage pathways and suggesting improved metabolic efficiency. AMP measurements suggested complementary interactions between the extracts. SP alone increased AMP levels at lower concentrations, whereas the combination produced lower AMP values than the untreated control. Although this pattern is consistent with improved cellular energy balance, the magnitude of the observed difference was relatively small and should therefore be interpreted cautiously pending verification of the statistical significance from the original analytical output. This suggests that the metabolic activity induced by one extract may be offset by improved energetic efficiency contributed by the other. Such coordination between glucose uptake, storage, and energy regulation reflects integrated metabolic control rather than isolated pathway activation [12,13,32]. These findings are consistent with the possibility of dual-pathway involvement, such as PI3K/Akt and AMPK signalling, although direct pathway activation was not assessed. Consequently, mechanistic interpretations should be considered provisional pending validation through targeted molecular studies.

### 4.4. Synergy Modelling and Methodological Robustness

Synergy assessment in botanical systems remains challenging because different analytical models are based on distinct assumptions regarding additivity and independent action. As a result, reliance on a single model may lead to biased interpretations. By demonstrating concordant interaction patterns across four independent models (Bliss, CI, Loewe, and ZIP), the present study provides evidence that the observed effects represent genuine biological interactions rather than artefacts of mathematical modelling [27,28]. The PG+SP combination exhibited concentration- and endpoint-dependent interactions ranging from additivity to synergy, indicating complementary rather than uniformly synergistic activity. Several endpoints demonstrated additive or near-additive interactions, whereas others exhibited clear synergistic responses, highlighting the context-dependent nature of botanical combination effects and reinforcing the importance of evaluating multiple biological endpoints using complementary interaction models. Notably, divergence between model families—where Loewe and ZIP indicated stronger synergy at lower concentrations and Bliss and CI at higher concentrations—likely reflects differences in sensitivity to dose–response curvature rather than true biological inconsistency [27,28]. We therefore interpret the synergy findings as complementary evidence of interaction rather than uniform demonstration of strong synergy.

### 4.5. Translational Relevance and Comparison with Conventional Therapy

The PG+SP combination achieved approximately 82–94% of metformin efficacy in these in vitro models, but this comparison requires caution given the differences between crude extracts and purified pharmacological agents [22,23]. The dose-sparing potential suggested by CI values (0.51–0.67) is theoretically attractive, though the non-linear dose–response relationship, with peak glucose uptake at the lowest concentration, which indicates that dose optimisation, not escalation, would be critical. The relationship between in vitro concentrations and achievable tissue levels following oral administration remains unknown.

Regarding toxicity, the combination preserved HepG2 viability above 89% at 500 µg/mL, substantially above the efficacious concentration range, suggesting a potentially favourable cytotoxicity profile in this hepatic model. However, this finding is limited to HepG2 cells; cytotoxicity in other cell types and under chronic exposure was not assessed. The cytoprotective synergy may be particularly relevant for T2DM patients with NAFLD, who represent a substantial proportion of the diabetic population and may have heightened susceptibility to hepatocellular stress [7,8], though this requires in vivo validation.

Potential herb–drug interactions with metformin, insulin, or sulphonylureas remain speculative but warrant future investigation, as does the possibility of CYP450 or P-glycoprotein modulation [22,23]. The African context is noteworthy, both plants are indigenous to regions with rising T2DM prevalence and limited access to conventional therapies [1,2,17]. But the current in vitro findings are insufficient to support clinical use. The tissue-selective responses observed suggest biological specificity that may be pharmacologically advantageous, though confirmation under physiological conditions is essential. These findings generate testable hypotheses for future investigation but should be viewed as a mechanistic foundation rather than evidence supporting clinical application. The relationship between the concentrations used in vitro and achievable tissue concentrations following oral administration remains unknown and will require pharmacokinetic evaluation.

### 4.6. Limitations and Future Directions

Several limitations should be considered. All experiments were conducted in vitro using HepG2 and C2C12 cell models, which do not fully replicate systemic physiology. The extracts were evaluated as crude aqueous preparations without standardisation against specific marker compounds, limiting the ability to attribute effects to individual constituents. Mechanistic interpretations remain inferential, as pathway-level validation was not performed, and pharmacokinetic properties were not investigated. Methodologically, the number of biological replicates was limited in some assays, C2C12 differentiation was confirmed morphologically rather than quantitatively [45,46], and metabolic measurements were not normalized to protein content. Future studies incorporating in vivo models, phytochemical standardisation, pharmacokinetic evaluation, and targeted molecular analyses are needed to validate these findings.

## 5. Conclusions

This study demonstrates that the combination of *Psidium guajava* and *Seriphium plumosum* modulates multiple parameters associated with glucose homeostasis in complementary hepatic and skeletal muscle cell models. The PG+SP combination exhibited concentration- and endpoint-dependent interactions that ranged from additivity to synergy, indicating complementary rather than uniformly synergistic biological activity. Across the experimental systems evaluated, combination treatment was associated with enhanced glucose uptake, increased glycogen accumulation, reduced GLUT2 protein expression, and alterations in AMP-related indices of cellular energy status. The consistency of interaction patterns observed across multiple analytical frameworks, including Bliss Independence, Combination Index (CI), Loewe Additivity, and Zero Interaction Potency (ZIP) models, strengthens confidence in the interpretation of the observed combination effects and highlights the value of multi-model approaches for assessing interactions within complex botanical preparations. Collectively, these findings provide mechanistic evidence that supports further investigation of the *P. guajava-S. plumosum* combination as a potential multi-target intervention for dysregulated glucose metabolism. Importantly, this study makes several novel contributions to the field. To the best of our knowledge, it represents the first experimental evaluation of the PG+SP combination in live-cell models, the first report describing SP-associated modulation of hepatic GLUT2 expression, and the first assessment of coordinated responses across both hepatic and skeletal muscle compartments for this botanical combination. Furthermore, the application of four independent synergy frameworks provides a more comprehensive characterisation of plant-plant interactions than approaches relying on a single interaction model. Nevertheless, these findings should be interpreted within the limitations of an exploratory in vitro investigation using crude aqueous extracts. The molecular pathways underlying the observed responses were not directly examined, and the relationship between the concentrations tested in vitro and achievable physiological exposure in vivo remains unknown. Consequently, further studies incorporating phytochemical standardisation, pharmacokinetic characterisation, molecular pathway validation, and whole-organism models will be required to determine whether the additive-to-synergistic interactions observed under controlled experimental conditions are maintained under physiological conditions. Overall, the present findings establish a scientific foundation for future investigations of the PG+SP combination and contribute to addressing the limited mechanistic evidence available for African polyherbal therapies in the context of type 2 diabetes mellitus. The coordinated responses observed across multiple metabolic endpoints support continued evaluation of this combination as a potential multi-target strategy for metabolic disease research while providing a framework for future in vivo validation and translational development.

## Figures and Tables

**Table 1:** Bliss Independence synergy analysis for the PG+SP combination in C2C12 skeletal muscle myoblasts.

Outcome Parameter	Concentration (µg/mL)	Observed Value ^a^	Expected Value ^b^	Bliss Score ^c^	Interpretation
**Glucose Uptake (%)**					
**Untreated control**	0	38.05 ± 0.15	—	—	—
	244 + 243	45.73	44.20	+1.53	Synergy
	488 + 486	44.74	43.45	+1.29	Synergy
	976 + 972	44.90	42.96	+1.94	Synergy
**Glycogen Content (×10^6^ RFU)**					
**Untreated control**	0	9.36 ± 0.12	—	—	—
	244 + 243	11.00	10.30	+0.70	Synergy
	488 + 486	10.70	10.43	+0.27	Additive
	976 + 972	11.00	10.55	+0.45	Synergy
**AMP Levels (RU) ^d^**					
**Untreated control**	0	3.87 ± 0.05	—	—	—
	244 + 243	0.19 e	−0.21	+0.40	Synergy
	488 + 486	0.26 e	−0.07	+0.33	Synergy
	976 + 972	0.14 e	−0.03	+0.17	Synergy

^a^ Observed values are mean ± SEM from three independent experiments (*n* = 3). ^b^ Expected values calculated as: Expected = Effect_PG + Effect_SP − Control value. ^c^ Bliss score interpretation thresholds (fractional scale): score > +0.10 = synergy; −0.10 to +0.10 = additive; score < −0.10 = antagonism. ^d^ For AMP analysis, values were transformed to ΔAMP (Control − AMP) to align with Bliss directionality. e Values shown are ΔAMP (Control − AMP) in RU.

**Table 2:** Estimated Loewe and ZIP Synergy Analysis for PG+SP Combination in C2C12 Myoblasts.

Outcome Parameter	PG+SP Concentration (µg/mL)	Observed Effect ^a^	Loewe Score ^b^	ZIP Score ^c^	Interpretation
**Glucose Uptake (%)**	244 + 243	45.73 ± 0.28	+3.2	+4.1	Mild synergy
	488 + 486	44.74 ± 0.26	+2.8	+3.5	Mild synergy
	976 + 972	44.90 ± 0.31	+5.1	+6.0	Moderate synergy
**Glycogen Content (×10^6^ RFU)**	244 + 243	11.00 ± 0.09	+6.7	+7.4	Moderate synergy
	488 + 486	10.70 ± 0.10	+2.1	+2.8	Additive
	976 + 972	11.00 ± 0.12	+4.8	+5.6	Moderate synergy
**ΔAMP Levels ^d^**	244 + 243	0.19	+4.5	+5.2	Moderate synergy
	488 + 486	0.26	+3.9	+4.4	Mild synergy
	976 + 972	0.14	+2.2	+2.7	Additive to mild synergy

^a^ Values represent mean ± SEM from three independent experiments. ^b^ Loewe additivity scores estimated from fixed-ratio concentration-response relationships. ^c^ ZIP synergy scores estimated using observed versus expected potency deviation analysis. ^d^ AMP values were transformed to ΔAMP (Control − AMP) prior to synergy modelling to maintain directional consistency, where higher values indicate improved cellular energy status.

## Data Availability

The original contributions presented in this study are included in the article/Supplementary Material. Further inquiries can be directed to the corresponding author.

## Supplementary Materials

The following supporting information can be downloaded at: https://www.mdpi.com/article/10.3390/nu18172920/s1, Figure S1, BPI UPLC-HRMS chromatograms of *Psidium guajava* and *Seriphium plumosum* aqueous extracts in positive ESI mode) and Tables S1 and S2 (tentative identification of compounds in negative ESI mode for *S. plumosum* and *P. guajava*, respectively).

## Abbreviations

Abbreviation	Full Term
PG	*Psidium guajava*
SP	*Seriphium plumosum*
T2DM	Type 2 Diabetes Mellitus
HepG2	Human hepatoma cell line
C2C12	Mouse skeletal muscle myoblast cell line
GLUT2	Glucose transporter type 2
GLUT4	Glucose transporter type 4
AMPK	AMP-activated protein kinase
PI3K	Phosphoinositide 3-kinase
Akt	Protein kinase B
HNF1α	Hepatocyte nuclear factor 1 alpha
NAFLD	Non-alcoholic fatty liver disease
CI	Combination Index
ZIP	Zero Interaction Potency
RFU	Relative fluorescence units
RU	Relative units
RIPA	Radioimmunoprecipitation assay buffer
TMB	Tetramethylbenzidine
HRP	Horseradish peroxidase
BCA	Bicinchoninic acid
Nrf2	Nuclear factor erythroid 2–related factor 2

## References

[1] International Diabetes Federation (2021). IDF Diabetes Atlas.

[2] Chikwati R., Jaff N., Ramsay M., Micklesfield L.K., Norris S.A., Seakamela K.P., Nonterah E.A., Agongo G., Mohamed S.F., Kisiangani I. (2025). Incident type 2 diabetes and its risk factors in men and women aged 40–60 years from four sub-Saharan African countries: Results from the AWI-Gen study. Lancet Glob. Health.

[3] DeFronzo R.A. (2009). From the triumvirate to the ominous octet: A new paradigm for the treatment of type 2 diabetes mellitus. Diabetes.

[4] Kahn S.E., Cooper M.E., Del Prato S. (2014). Pathophysiology and treatment of type 2 diabetes: Perspectives on the past, present, and future. Lancet.

[5] Thembane N., Hlatshwayo S., Mhlungu S., Sithole S., Ngubane P., Ngcobo M., Gqaleni N. (2024). Review on the anti-hyperglycemic potential of *Psidium guajava* and *Seriphium plumosum* L.. Plants.

[6] Moore M.C., Coate K.C., Winnick J.J., An Z., Cherrington A.D. (2012). Regulation of hepatic glucose uptake and storage in vivo. Compr. Physiol..

[7] Rizza R.A. (2010). Pathogenesis of fasting and postprandial hyperglycemia in type 2 diabetes: Implications for therapy. Diabetes.

[8] Samuel V.T., Shulman G.I. (2016). The pathogenesis of insulin resistance: Integrating signaling pathways and substrate flux. J. Clin. Investig..

[9] Thiebaud D., Jacot E., DeFronzo R.A., Maeder E., Jequier E., Felber J.-P. (1982). The effect of graded doses of insulin on total glucose uptake, glucose oxidation, and glucose storage in man. Diabetes.

[10] Baron A.D., Brechtel G., Wallace P., Edelman S.V. (1988). Rates and tissue sites of non-insulin- and insulin-mediated glucose uptake in humans. Am. J. Physiol..

[11] Shepherd P.R., Kahn B.B. (1999). Glucose transporters and insulin action: Implications for insulin resistance and diabetes mellitus. N. Engl. J. Med..

[12] Abdul-Ghani M.A., DeFronzo R.A. (2010). Pathogenesis of insulin resistance in skeletal muscle. J. Biomed. Biotechnol..

[13] Zhou G., Myers R., Li Y., Chen Y., Shen X., Fenyk-Melody J., Wu M., Ventre J., Doebber T., Fujii N. (2001). Role of AMP-activated protein kinase in mechanism of metformin action. J. Clin. Investig..

[14] Foretz M., Guigas B., Bertrand L., Pollak M., Viollet B. (2014). Metformin: From mechanisms of action to therapies. Cell Metab..

[15] Nathan D.M. (2014). The diabetes control and complications trial/epidemiology of diabetes interventions and complications study at 30 years: Overview. Diabetes Care.

[16] Bailey C.J., Turner R.C. (1996). Metformin. N. Engl. J. Med..

[17] Beran D., Yudkin J.S. (2006). Diabetes care in sub-Saharan Africa. Lancet.

[18] Inzucchi S.E., Bergenstal R.M., Buse J.B., Diamant M., Ferrannini E., Nauck M., Peters A.L., Tsapas A., Wender R., Matthews D.R. (2015). Management of Hyperglycemia in Type 2 Diabetes, 2015: A Patient-Centered Approach: Update to a Position Statement of the American Diabetes Association and the European Association for the Study of Diabetes. Diabetes Care.

[19] Wagner H., Ulrich-Merzenich G. (2009). Synergy research: Approaching a new generation of phytopharmaceuticals. Phytomedicine.

[20] Jia J., Zhu F., Ma X., Cao Z., Li Y., Chen Y. (2009). Mechanisms of drug combinations: Interaction and network perspectives. Nat. Rev. Drug Discov..

[21] Chou T.-C. (2006). Theoretical basis, experimental design, and computerized simulation of synergism and antagonism in drug combination studies. Pharmacol. Rev..

[22] Caesar L.K., Cech N.B. (2019). Synergy and antagonism in natural product extracts. Nat. Prod. Rep..

[23] Chou T.-C. (2010). Drug combination studies and their synergy quantification using the Chou–Talalay method. Cancer Res..

[24] Bliss C.I. (1939). The toxicity of poisons applied jointly. Ann. Appl. Biol..

[25] Loewe S. (1953). The problem of synergism and antagonism of combined drugs. Arzneimittelforschung.

[26] Yadav B., Wennerberg K., Aittokallio T., Tang J. (2015). Searching for drug synergy in complex dose–response landscapes using an interaction potency model. Comput. Struct. Biotechnol. J..

[27] Foucquier J., Guedj M. (2015). Analysis of drug combinations: Current methodological landscape. Pharmacol. Res. Perspect..

[28] Greco W.R., Bravo G., Parsons J.C. (1995). The search for synergy: A critical review from a response surface perspective. Pharmacol. Rev..

[29] Abruscato G., Tarantino R., Mauro M., Chiarelli R., Vizzini A., Arizza V., Vazzana M., Luparello C. (2024). Modulation of glucose consumption and uptake in HepG2 cells by aqueous extracts from the coelomic fluid of the edible *Holothuria tubulosa* sea cucumber. Biology.

[30] Roy J.R., Janaki C.S., Jayaraman S., Periyasamy V., Balaji T., Vijayamalathi M., Veeraraghavan V.P. (2022). *Carica papaya* reduces muscle insulin resistance via IR/GLUT4 mediated signaling mechanisms in high-fat diet and streptozotocin-induced type 2 diabetic rats. Antioxidants.

[31] Narvaez J.J., Guerrero-Analco J.A., Monribot-Villanueva J.L., Chan-Zapata I., Campos M.R. (2025). Metabolic profile of *Psidium guajava* L. fruits and their inhibitory properties on α-amylase and α-glucosidase. Fitoterapia.

[32] Tain Y.-L., Lin Y.-J., Sheen J.-M., Lin I.-C., Yu H.-R., Huang L.-T., Hsu C.-N. (2017). Resveratrol prevents the combined maternal plus postweaning high-fat diets-induced hypertension in male offspring. J. Nutr. Biochem..

[33] Díaz-de-Cerio E., Pasini F., Verardo V., Fernández-Gutiérrez A., Segura-Carretero A., Caboni M.F. (2017). Phenolic compounds in *Psidium guajava*. Food Res. Int..

[34] Salem M.Z., Al-Kubeisi A.K., Ashmawy A.M., Mansour M., Aboelela S., Elshaer M.A., Selim S. (2026). Extracts from Some Tree Pruning Residues and their Cytotoxicity on the HepG2 Cell Line Using the Sulforhodamine-B Assay and their Antifungal Activity. BioResources.

[35] Van Wyk B.E. (2015). A review of commercially important African medicinal plants. J. Ethnopharmacol..

[36] Beseni B.K., Bagla V.P., Njanje I., Matsebatlela T.M., Mampuru L., Mokgotho M.P. (2017). Antioxidant, Antiglycation, and Hypoglycaemic Effect of *Seriphium plumosum* Crude Plant Extracts. Evid.-Based Complement. Altern. Med..

[37] Mueckler M., Thorens B. (2013). The SLC2 (GLUT) family of membrane transporters. Mol. Asp. Med..

[38] Thorens B. (2015). GLUT2, glucose sensing and glucose homeostasis. Mol. Metab..

[39] Karim S., Adams D.H., Lalor P.F. (2012). Hepatic glucose transporter distribution. World J. Gastroenterol..

[40] Postic C., Dentin R., Girard J. (2004). Role of the liver in the control of carbohydrate and lipid homeostasis. Diabetes Metab..

[41] Herman M.A., Kahn B.B. (2006). Glucose transport and sensing in the maintenance of glucose homeostasis and metabolic harmony. J. Clin. Investell..

[42] Chandran U., Mehendale N., Patil S., Choudhary R., Patwardhan B. (2017). Network pharmacology. J. Ayurveda Integr. Med..

[43] Donato M.T., Tolosa L., Gómez-Lechón M.J. (2015). HepG2 characterization. Methods Mol. Biol..

[44] Hricovíniová J., Ševčovičová A., Hricovíniová Z. (2020). Evaluation of the genotoxic, DNA-protective and antioxidant profile of synthetic alkyl gallates and gallotannins using in vitro assays. Toxicol. In Vitro.

[45] Yaffe D., Saxel O. (1977). Serial passaging and differentiation of myogenic cells isolated from dystrophic mouse muscle. Nature.

[46] Burattini S., Ferri P., Battistelli M., Curci R., Luchetti F., Falcieri E. (2004). C2C12 murine myoblasts as a model of skeletal muscle development: Morpho-functional characterization. Eur. J. Histochem..

[47] International Committee of Medical Journal Editors Recommendations for the Conduct, Reporting, Editing, and Publication of Scholarly Work in Medical Journals. https://www.icmje.org/.

[48] National Center for Biotechnology Information PubMed Database. https://pubmed.ncbi.nlm.nih.gov/.

[49] He F., Su S., Song R., Li Y., Zou L., Li Z., Xiao Y., Hou A., Li K., Wang Y. (2025). Synergistic anti-type 2 diabetes effects of *Morus alba* and *Siraitia grosvenorii*. Antioxidants.

[50] Tanaka T., Ishida N., Ishimatsu M., Nonaka G.I., Nishioka I. (1992). Tannins and related compounds. CXVI. Six new complex tannins, guajavins, psidinins and psiguavin from the bark of *Psidium guajava* L.. Chem. Pharm. Bull..

[51] Yang X.L., Hsieh K.L., Liu J.K. (2007). Guajadial: An unusual meroterpenoid from guava leaves *Psidium guajava*. Org. Lett..

[52] (2025). Waters_Connect UNIFI® Scientific Information System.

[53] Abbas A., Naqvi S.A.R., Rasool M.H., Noureen A., Mubarik M.S., Tareen R.B. (2021). Phytochemical analysis, antioxidant and antimicrobial screening of *Seriphidium oliverianum* plant extracts. Dose-Response.

[54] Gutiérrez R.M.P., Mitchell S., Solis R.V. (2008). *Psidium guajava*: A review of its traditional uses, phytochemistry and pharmacology. J. Ethnopharmacol..

[55] Huynh H.D., Nargotra P., Wang H.M.D., Shieh C.J., Liu Y.C., Kuo C.H. (2025). Bioactive compounds from guava leaves (*Psidium guajava* L.): Characterization, biological activity, synergistic effects, and technological applications. Molecules.

