# Supplementary material for: A Combination of Psidium guajava and Seriphium plumosum Modulates Glucose Metabolism and Reduces Hepatic GLUT2 Expression Through Additive-to-Synergistic Interactions
Source: Nutrients. 2026 Sep 6;18(17):2920. doi: 10.3390/nu18172920 (PMC13567443; doi:10.3390/nu18172920)
Supplement: Supplementary file 1 [file nutrients-18-02920-s001.zip › nutrients-4473837-supplementary.pdf]

Supplementary Materials: Chemical profiling of the extracts

Chemical Profiling of the Extracts

This supplementary document provides supporting chromatographic and compound identification data for the aqueous extracts of *Psidium guajava* (PG) and *Seriphium plumosum* (SP) analysed by UPLC-PDA-HRMS.

Figure S1 presents base peak intensity (BPI) chromatograms in positive ESI mode for both extracts (S1C and S1D), complementing the negative mode chromatograms presented in Figure 1 of the main text (Figure 1A and 1B). Blank chromatograms (S1A and S1B) are included as negative controls to confirm the absence of background contamination or column bleed, consistent with standard UPLC-HRMS quality assurance practices.

Tables S1 and S2 provide detailed compound annotations for SP and PG, respectively. Compounds with documented antidiabetic, glucose-lowering, or insulin-sensitising activity are indicated in **bold** and discussed in the main text (Sections 4.2 and 4.3).

A

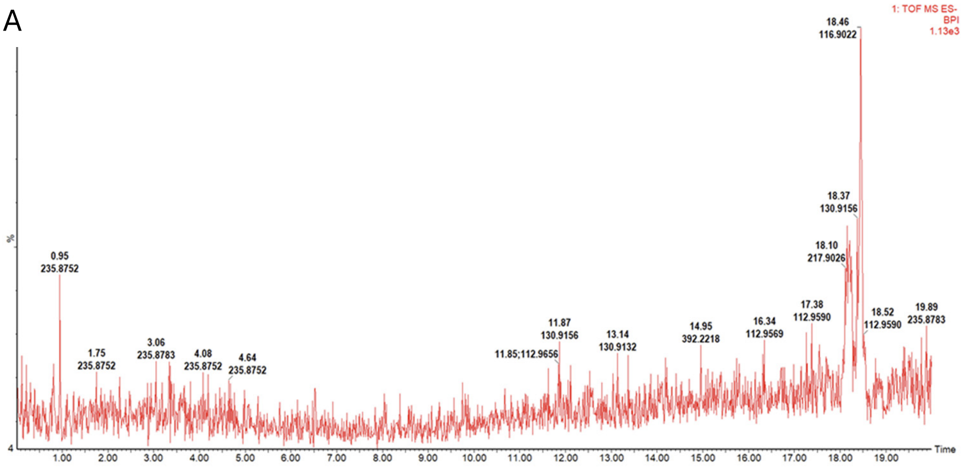

B

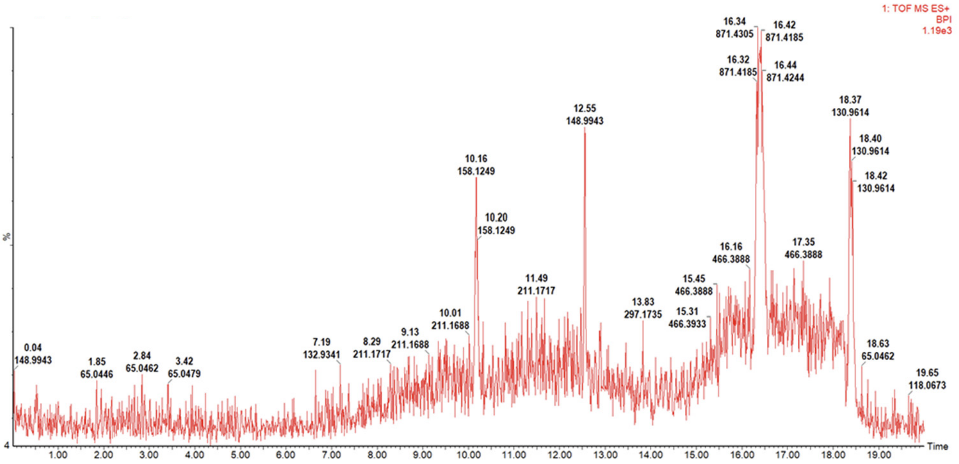

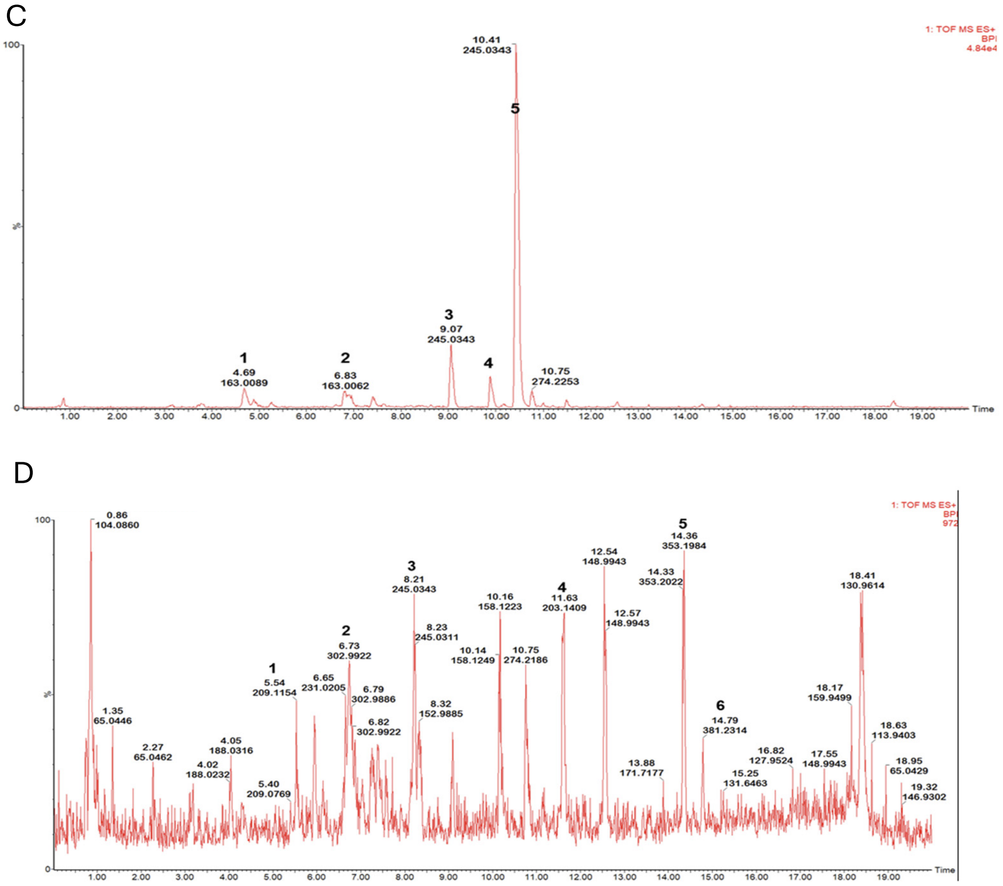

Figure S1. Base peak intensity (BPI) UPLC-HRMS chromatograms of (A) blank (mobile phase) in negative ESI mode, (B) blank (mobile phase) in positive ESI mode, (C) *S. plumosum* (VJM-I-150A) aqueous extract in positive ESI mode, and (D) *P. guajava* (VJM-I-150B) aqueous extract in positive ESI mode.

Table S1. Tentative identification of compounds in the aqueous extract of *S. plumosum* (VJM-I-150A) by UPLC-HRMS in negative ESI mode.

Peak	RT (min)	*m/z*	Adducts	Mass error (ppm)	Formula of tentative compound	Calculated accurate mass	Tentative compound	Structure	Ref
1	1.35	191.0189	$[M\text{-}H]^-$	-4.1	$C_6H_8O_7$	192.0270	**Citric acid**	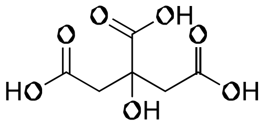	[50]
2	3.30	153.0181	$[M\text{-}H]^-$	-7.9	$C_7H_6O_4$	154.0266	**2,4-dihydroxybenzoic acid**	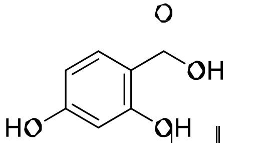	[50]
3	4.58	349.0588	$[M+HCO_2]^-$	6.5	$C_{15}H_{12}O_7$	304.0583	**Taxifolin**	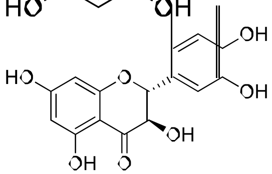	[50]
4	4.71	353.0863	$[M\text{-}H]^-$	-4.3	$C_{16}H_{18}O_9$	354.0951	**4-Caffeoylquinic acid**	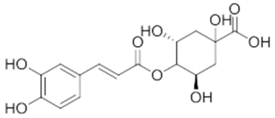	[50]
5	4.99	179.0339	$[M\text{-}H]^-$	-5.9	$C_9H_8O_4$	180.0423	**Caffeic acid**	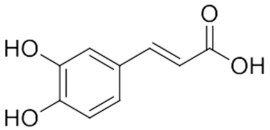	[50,53]
6	5.26	515.1183	$[M\text{-}H]^-$	-1.4	$C_{25}H_{24}O_{12}$	516.1268	**1,5-Dicaffeoylquinic acid**	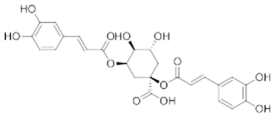	[50]
7	6.79	515.1194	$[M\text{-}H]^-$	-0.1	$C_{25}H_{24}O_{12}$	516.1268	**1,5-Dicaffeoylquinic acid(isomer)**	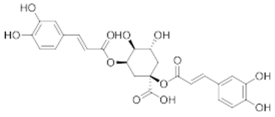	[50]

8	7.38	515.1194	$[M-H]^-$	-0.7	$C_{25}H_{24}O_{12}$	516.1268	**1,5-Dicaffeoylquinic acid(Isomer)**	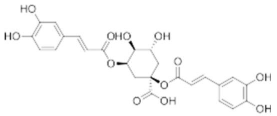	[50]
9	7.63	261.0760	$[M-HCO_2]^-$	-3.1	$C_{15}H_{14}O_7$	306.0740	**(-)-Epigallocatechin**	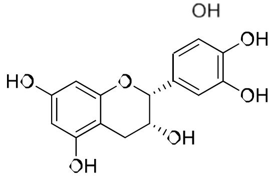	[50]
10	8.21	329.1012	$[M-HCO_2]^-$	-5.7	$C_{19}H_{18}O_8$	374.1002	**Chrysosplenetin**	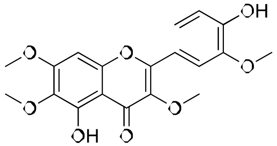	[50]
11	10.01	395.1364	$[M+CH_3CO_2]^-$	-2.5	$C_{20}H_{18}NO_4^+$	336.1236	**Berberine**	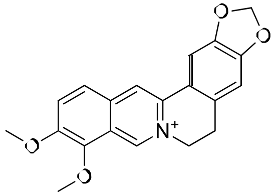	[50,53]
12	10.28	427.1400	$[M+CH_3CO_2]^-$	0.3	$C_{21}H_{20}O_6$	368.1260	**Curcumin**	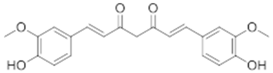	[50]
13	10.68	429.1559	$[M-H]^-$	0.9	$C_{23}H_{26}O_8$	430.1628	**Episesartemin**	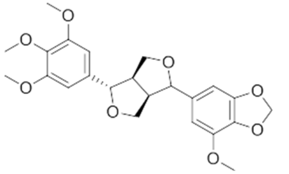	[50]
14	12.16	311.2241	$[M-H]^-$	6.1	$C_{18}H_{32}O_4$	312.2300	Unidentified	Unidentified	-
15	12.61	293.2122	$[M-H]^-$	1.7	$C_{18}H_{30}O_3$	294.2195	Unidentified	Unidentified	-

*Compounds with documented antidiabetic, glucose-lowering, or insulin-sensitising activity are indicated in **bold**. These include berberine (potent AMPK activator), curcumin (anti-inflammatory, insulin sensitisation), caffeoylquinic acid derivatives (α-glucosidase inhibitors), caffeic acid (antioxidant, insulin sensitivity), (-)-epigallocatechin (AMPK modulation, β-cell protection), and flavonoids (taxifolin, chrysosplenetin) with documented metabolic benefits [50,53]*

Table S2. Tentative identification of compounds in the aqueous extract of *P. guajava* (VJM-I-150B) by UPLC-HRMS in negative ESI mode.

Peak	RT (min)	*m/z*	Adducts	Mass error (ppm)	Formula of tentative compound	Calculated accurate mass	Tentative compound	Structure	Ref
1	1.91	168.0193	$[M-H]^-$	-6.3	$C_7H_6O_5$	170.0215	**Gallic acid**	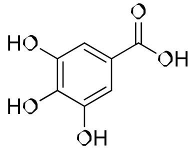	[50,54,55]
2	3.93	577.1351	$[M-H]^-$	0.0	$C_{30}H_{26}O_{12}$	578.142	**Procyanidin B1**	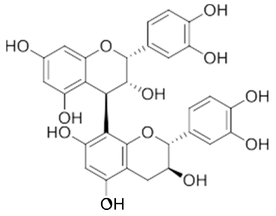	[50,55]
3	4.30	289.0762	$[M-H]^-$	17.3	$C_{15}H_{14}O_6$	290.0790	**Catechin**	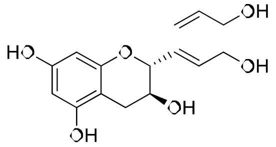	[50,55]
4	5.39	305.0685	$[M-H]^-$	7.9	$C_{15}H_{14}O_7$	306.0740	**Epigallocatechin**	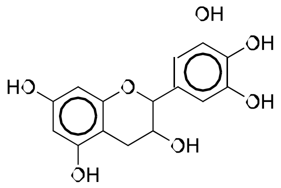	[50,55]
5	6.74	380.9909	$[M-H]^-$	-8.4	$C_{11}H_{10}O_{15}$	382.0020	Unidentified	Unidentified	-

6	8.36	557.1288	$[M-H]^-$	-1.3	$C_{27}H_{26}O_{13}$	558.1373	**Kaempferol-3-O-(2,3,4-tri-O-acetyl-α-L-rhamnopyranoside)**	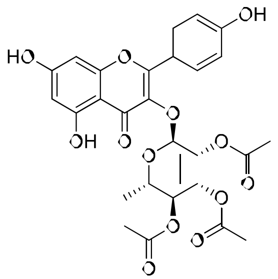	[50]
7	11.67	549.3437	$[M+HCO_2]^-$	0.7	$C_{30}H_{48}O_6$	504.3451	Arjugenin	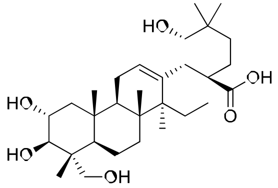	[50]
8	11.94	271.0603	$[M-HCO_2]^-$	-3.2	$C_{16}H_{12}O_7$	316.0583	**Rhamnetin**	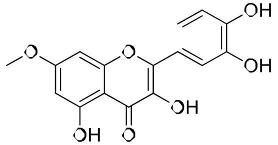	[50]
9	13.81	491.2443	$[M-H]^-$	-3.9	$C_{30}H_{36}O_6$	492.2571	Unidentified	Unidentified	-
10	14.48	491.2425	$[M-H]^-$	-1.8	$C_{30}H_{36}O_6$	492.2512	Unidentified	Unidentified	-
11	16.10	473.2318	$[M-H]^-$	-2.1	$C_{30}H_{34}O_5$	474.2406	(1S,4S,7R,11R,12S)-14,16-dihydroxy-1,5,5-trimethyl-8-methylidene-12-phenyl-19-oxatetracyclo[9.8.0.$0^{4,7}$.$0^{13,18}$]nonadeca-13,15,17-triene-15,17-dicarbaldehyde	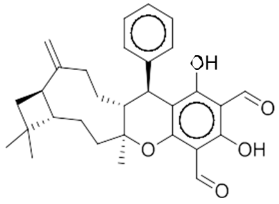	[50,52]

Compounds with documented antidiabetic, glucose-lowering, or insulin-sensitising activity are indicated in ***bold****. These include gallic acid (α-glucosidase inhibition, insulin secretion enhancement), catechin and epigallocatechin (AMPK activators, SGLT2/DPP-4 inhibition), procyanidin B1 (insulin sensitivity improvement), kaempferol derivatives (insulin sensitivity), and rhamnetin (flavonoid with glucose-lowering properties) [50,54,55].*
